# Celebrating 30 years of science from the James Clerk Maxwell Telescope

**DOI:** 10.1098/rsos.170754

**Published:** 2017-09-06

**Authors:** Ian Robson, Wayne S. Holland, Per Friberg

**Affiliations:** 1 UK Astronomy Technology Centre, Royal Observatory, Blackford Hill, Edinburgh EH9 3HJ, UK; 2 Institute for Astronomy, University of Edinburgh, Royal Observatory, Blackford Hill, Edinburgh EH9 3HJ, UK; 3 East Asian Observatory, 660 N. A‘ohōkū Place, University Park, Hilo, HI 96720, USA

**Keywords:** galaxies, stars, planets

## Abstract

The James Clerk Maxwell Telescope (JCMT) has been the world’s most successful single-dish telescope at submillimetre wavelengths since it began operations in 1987. From the pioneering days of single-element photometers and mixers, through to the state-of-the-art imaging and spectroscopic cameras, the JCMT has been associated with a number of major scientific discoveries. Famous for the discovery of ‘SCUBA’ galaxies, which are responsible for a large fraction of the far-infrared background, the JCMT has pushed the sensitivity limits arguably more than any other facility in this most difficult of wavebands in which to observe. Closer to home, the first images of huge discs of cool debris around nearby stars gave us clues to the evolution of planetary systems, further evidence of the importance of studying astrophysics in the submillimetre region. Now approaching the 30th anniversary of the first observations, the telescope continues to carry out unique and innovative science. In this review article, we look back on some of the major scientific highlights from the past 30 years.

## 1. Introduction and overview

**Figure 1.**
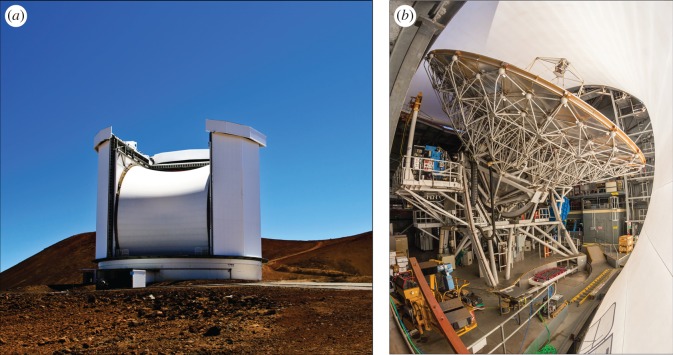
(*a*) The JCMT in its usual mode of operation with a protective membrane in place (courtesy of Royal Observatory Edinburgh). (*b*) Inside the enclosure showing the main antenna and secondary mirror unit. The HARP heterodyne array is seen on the platform to the left and SCUBA-2 on the right side (courtesy of East Asian Observatory).

The James Clerk Maxwell Telescope (JCMT) [1] (figure 1) has been the world’s premier single-dish submillimetre telescope since its opening in 1987. At 13 425 ft above sea level on Mauna Kea in Hawaii, it has the benefit of being above much of the water vapour that restricts ground-based, submillimetre astronomy to a few narrow ‘windows’ through which observations are possible. It was a purpose-built facility with a 15 m diameter, high-surface-accuracy primary mirror that feeds the incoming radiation to a receiver cabin at the Cassegrain focus behind the main dish and also to two Nasmyth platforms at each side of the elevation bearings.

The scientific output of any astronomical facility is a combination of many factors: the intrinsic efficiency of the design, location and weather, instrumentation capability and reliability, operability and the effectiveness of the data-reduction software. Throughout the life of the JCMT, the observatory strove to provide the world’s best instrumentation, improve the reliability of the facility and to maximize the scientific output for the funding agencies and astronomical community through science-ranked, weather-dependent, queue-based flexible scheduling of observations. Indeed, the three funding agencies of the UK (55%), Canada (25%) and The Netherlands (20%) deserve credit for their continued support of the developments of the facility and instrumentation even in times of severe financial pressure on home budgets.

This article discusses the scientific output of the JCMT, doing so through the eyes of the instrumentation suite as this provides one of the most coherent ways of seeing how technology has driven the scientific capability and new discoveries emanating from the facility. We have subdivided this treatment into four sections: the three continuum instrument epochs of UKT14, SCUBA, SCUBA-2 and the heterodyne spectrometers, culminating in HARP. Within these sections the science output is naturally grouped into astronomical themes. While it has not been possible to include all the science from the 30 years of operations, the science highlights readily stand out.

In terms of a timeline, the story can be broken down into some very clear regimes. In the continuum, we begin with the single-pixel bolometer UKT14 [2], the only ‘common-user’ submillimetre photometer in operation in the decade before the arrival of SCUBA in 1996. SCUBA, the Submillimetre Common-User Bolometer Array [3], was the world’s first submillimetre ‘camera’ with 128 pixels in two arrays, one operating at a primary wavelength of 850 μm with 37 pixels and one at 450 μm with 91 pixels. It is not an overstatement to say that SCUBA brought about the ‘submillimetre revolution’ in astronomy and the science highlights from this instrument are described in §3. The outstanding success of SCUBA immediately showed the need for a next-generation camera, one with many more pixels to provide a larger field-of-view and with improved sensitivity. This resulted in SCUBA-2 [4], a revolutionary instrument that was fraught with technological challenges. Nevertheless, it became operational on the telescope in 2011, and with over 5000 pixels at each of the main SCUBA wavelengths, it heralded the onset of large-scale mapping of the submillimetre sky. SCUBA-2 continues in operation to this day and §4 describes the major inroads of science this instrument has brought, moving from the snapshots of discrete objects or mapping tiny areas of sky, to large-scale imaging, resulting in statistically significant samples of objects and addressing evolution across many scale sizes and cosmological timescales.

In terms of heterodyne spectroscopy, the JCMT had a slower start and went through a number of iterations of single- and dual-pixel instruments operating in most of the submillimetre and near-millimetre atmospheric windows. The most successful of these was the 350 GHz (850 μm) receiver RxB3 [5,6], a dual-channel instrument receiving orthogonal linear polarizations from the same position on the sky, and which operated on the telescope between 1997 and 2006. The Digital Autocorrelating Spectrometer (DAS) [7] was used in conjunction with many of the early heterodyne mixers. The arrival of the 350 GHz 16-element Heterodyne Array Receiver Programme (HARP) receiver [8] heralded the ability to carry out high-resolution spectroscopy over large areas of sky. This instrument was introduced in 2007 and came with a new digital spectral correlator, the Auto Correlation Spectrometer Imaging System (ACSIS) [8]. The heterodyne suite of instruments has proved to be very successful over the years and some of the major science results are described in §5.

In addition to the facility instruments, the JCMT was open to visiting instruments as approved by the Telescope Allocation Panel. These included: a 345 GHz SIS receiver from Ed Sutton’s group at UC Berkeley [9], at a time when the JCMT only had Schottky diode receivers; a high-frequency 600 GHz and 800 GHz receiver (Receiver G) from Reinhard Genzel’s group at MPIA Bonn [10,11]; a Fourier Transform Spectrometer from David Naylor’s group at Lethbridge [12]; the South Pole Imaging Fabry-Pérot Instrument (SPIFI) [13] from Gordon Stacey’s group at Cornell; and AzTEC, a 144 pixel bolometer camera from Grant Wilson’s group at the University of Massachusetts (Amherst) [14]. Science highlights from some of these instruments are included in §§3 and 5.

The eventual move to flexible scheduling of observations, based on the science priority of the proposal and the weather at the telescope, led to an enormous increase in productivity and in subsequent scientific impact of the facility [15]. Although flexible scheduling was somewhat resisted by the users at the start, the eventual implementation meant that no longer were top-ranked proposals at the risk of being blighted by poor weather that happened to coincide with the fixed schedule of their observing run, but they would be undertaken throughout the semester when the weather was best suited to the scientific requirements. The JCMT was one of the first observatories to bring about this innovation and was a major operational advantage for SCUBA and the instruments that followed. Finally, the importance of an easily accessible and user-friendly archive was duly recognized, particularly when the volume of data being generated increased significantly (e.g. with the introduction of SCUBA). This led to the creation of the JCMT Science Archive (JSA) [16], hosted at the Canadian Data Archive Centre [17]. The JSA is designed to increase the productivity of the telescope by making not only the raw data, but also science-quality reduced images available to the JCMT and wider astronomy communities. This will allow the astronomers of the future to interrogate the data to explore, for example, time-dependent phenomena over the lifetime of the telescope.

## 2. Scientific results from UKT14

Although only a single-pixel device, UKT14 came with many improvements over its predecessors. It was constructed by the Royal Observatory Edinburgh and from the start it was designed as a ‘common-user’ instrument and crucially came with a user-friendly data reduction software suite. It was originally designed for and operated on the United Kingdom Infrared Telescope (UKIRT) but when moved to the JCMT brought more than a 100 times increase in sensitivity over UKIRT along with an increase of a factor of 4 in angular resolution, with beam sizes (full-width at half-maximum) of 14 arcsecs at 850 μm and 6–7 arcsecs at 350/450 μm. The instrument had very carefully designed optics to minimize stray radiation and a range of filters to select the atmospheric ‘windows’ allowing photometric observations to be made from 2 mm to 350 μm. This was very important for studies of spectral energy distributions, even though the most often used filters tended to be in the most stable windows in the submillimetre region at 800 and 450 μm. It was a very sensitive photometer, the composite germanium bolometer being cooled to 0.35 K by liquid He^3^, and capable of detecting point sources down to a level of approximately 8 mJy at 800 μm in 1 h of observing time. UKT14 turned out to be critically important: it was state-of-the-art in the early days of the JCMT; it opened up a range of new science ventures for submillimetre study; but perhaps most importantly, it introduced a whole new generation of astronomers to the field, many of whom were not necessarily submillimetre astronomy experts. Indeed, these were the same astronomers who would go on to make revolutionary discoveries with the introduction of SCUBA.

The science output from UKT14 was indeed huge, both in extent and depth. According to the JCMT Annual Reports, over 180 papers in refereed journals contained data from UKT14 before it was superseded by SCUBA in 1996. This is a staggering 44% of the total number of JCMT refereed papers over the same period, showing the dominance of UKT14 and continuum science in the first decade of the JCMT. The science topics ranged from observations of comets in the Solar System to high-redshift galaxies, mostly detecting the thermal emission from cold dust grains. There was a long-standing and very successful programme of monitoring the flaring emission from blazars, however, which originated from non-thermal emission from relativistic electrons. Because ground-based submillimetre astronomy was still in its infancy, UKT14 was also used extensively for assessing observing techniques, studying the atmospheric extinction and identifying calibration sources, all of which would provide the sound basis for visiting astronomers to build on [18]. In compiling examples of UKT14 science, we have tried to show the breadth of the different astrophysical topics opened up by this remarkably versatile instrument, and so present a wide but relatively shallow selection of topics, although selected mainly through citation indexes.

### 2.1. Solar System studies

One of the early observations with UKT14 was the first detection of a comet at submillimetre wavelengths in 1989 by Jewitt & Luu [19]. They found that the emission of comet P/Brorsen-Metcalf could be modelled by a population of transient, large grains with a total mass of approximately 10^9^ kg, which could have been produced by some form of breakdown of part of the refractory mantle of the comet. Later observations of the comet Hyakutake in 1996 by Jewitt & Matthews [20] found that from 1.1 mm to 350 μm the emission can be described as thermal emission from large (approx. 1 mm) dust grains in the coma and a resulting total mass of around 2×10^9^ kg. The spectral index indicates that the opacity factor is similar to that found in the circumstellar discs of young stars. Remarkably, a small map was made at 800 μm, which showed that the emission was consistent with the steady emission of solid particles from the cometary nucleus on timescales less than 1 day. A major study by Redman *et al*. [21] of the asteroid 4-Vesta in 1989 showed that the submillimetre emission might originate from a form of dusty, porous regolith. Furthermore, unlike the single-peaked rotational light curve in the optical, the millimetre light curve was seen to be double-peaked, indicating that it was most probably dominated by the triaxial shape of the asteroid. A major investigation was undertaken by Griffin & Orton [22] who measured the emission from Uranus and Neptune from 2 mm to 350 μm. These precise data allowed the brightness temperatures of the planetary atmospheres to be calculated with greater accuracy (with uncertainties of less than 2 K) based on data from Mars, the primary calibration source at submillimetre wavelengths. These new values enabled Uranus to become a valuable calibrator for submillimetre observations, both for ground- and space-based facilities. Addressing both calibration purposes and intrinsic properties of asteroids, Müller & Lagerros [23] used JSA data on 1-Ceres, 2-Pallas, 4-Vesta, 532-Herculina, 10-Hygiea, 106-Dione and 313-Chaldaea to determine thermal models for the asteroids as well as defining new far-infrared (far-IR) photometric standards to be used by the ISOPHOT instrument on the *ISO* satellite. This demonstrated the value and accessibility of the JSA. The work also built on a programme of observations that produced a major publication by Redman *et al*. [24] in which the spectral energy distribution was obtained for seven asteroids, five of which were non-metallic and two were metallic. The data showed that there was a notable range of physical properties of the surfaces, even for the non-metallic bodies.

To conclude this section, Stern *et al*. [25] succeeded where many had previously failed and detected Pluto at 1.3 mm and 800 μm, deducing a surface temperature of 30–44 K with a most probable range of 35–37 K. This range is significantly lower than had been predicted from radiative equilibrium models and from other observations and showed that the methane features in Pluto’s spectrum were from solid, rather than gas-phase, absorptions, demonstrating that Pluto’s atmosphere is dominated by nitrogen or carbon monoxide rather than methane.

### 2.2. Star formation

**Figure 2.**
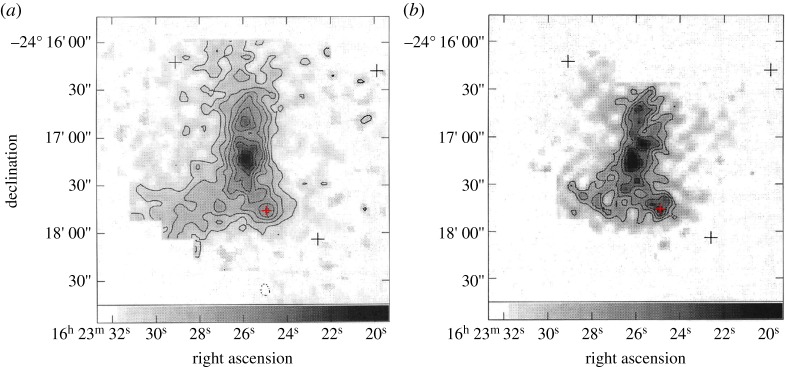
The *ρ* Ophiuchus A molecular cloud at 800 μm (*a*) and 450 μm (*b*). The red cross towards the lower right of the cloud extent marks the position of the Class 0 protostar, VLA 1623. Adapted from André *et al*. [26].

The very early stages of star formation, detecting the emission from cold dust, turned out to be one of the key areas for UKT14 study and was one of the most popular series of targets resulting in many publications over the period. One of the most spectacular sets of observations and, indeed, the most cited UKT14 result (with over 900 citations) came from maps by André *et al*. [26] of the *ρ* Ophiuchus molecular cloud in which the protostellar source VLA 1623 was proposed as a new category, ‘Class 0’, as the earliest phase in the star formation sequence. The observations of the core of the cloud at 800 and 450 μm took 18 h of integration over three nights of excellent and stable weather. The results are shown in figure 2. The emission shows four compact clumps with masses approximately 1 solar mass (M_⊙_) embedded in a ridge of approximately 15 M_⊙_. VLA 1623 is the coldest clump with a temperature estimated to be 15–20 K, appearing to have no central heat source and not detected by the *IRAS* satellite. The mass was estimated to be 0.6 M_⊙_ with a luminosity of around the same as the Sun. These observations showed the potential of the submillimetre for uniquely being able to study the earliest phases of star formation and this paper was a landmark in the field. We return to the study of molecular clouds and protostars with the advent of SCUBA (§§3.5 and 3.6) and SCUBA-2 (§§4.1.2 and 4.2.5).

The observations of *ρ* Oph were rapidly followed up with the next most cited UKT14 paper (close to 400 citations), in which Ward-Thompson *et al*. [27] made observations of 21 cold molecular cores in dark clouds with no infrared source; the so-called ‘starless cores’. These clumps have insufficient bolometric luminosity to be typical of a ‘Class I’ protostar and a crucial discovery was that these cores differed from those that had an *IRAS* far-IR detection in that they are all more diffuse and less centrally peaked. The clumps had densities 10^5^ to 10^6^ *cm*^−3^ but the density profile was inconsistent with the *r*^−2^ or *r*^−3/2^ profiles predicted by standard theory and instead were more consistent with magnetic support. The authors concluded that these submillimetre bright, dark cores are indeed pre-protostellar and are in the very earliest stages prior to protostellar collapse.

Saraceno *et al.* [28] conducted a series of observations of a sample of 45 Class I and Class 0 young stellar objects (YSOs) at 1.3 mm using UKT14 and the Swedish-ESO Submillimetre Telescope (SEST) and made a number of important conclusions regarding their evolutionary sequencing. These included that the evolution of a protostar was mainly controlled by the mass of both the central object and circumstellar material and that the Class 0 sources were indeed the earliest stages of star formation yet observed. Also, the Class I sources showing outflow had dynamical timescales exceeding 10 000 years and that they were probably in the deuterium-burning phase where they spend most of their lifetime accreting material. On the other hand, the Class I sources with no outflow behave like Class II sources with no outflow, and the authors suggest that these are most probably Class II sources suffering high extinction from foreground emission. Further examples of early phases of star formation can be found in the search for protostellar cores in Bok globules [29] and in *IRAS* sources [30,31].

Although the Orion complex of clouds and star formation were a notable source of study, perhaps surprisingly they hardly feature in the highly rated citations of UKT14. Chini *et al*. [32] produced probably the most definitive study of submillimetre emission from OMC-2 and OMC-3, detecting six probable Class 0 protostars as well as describing the general dust morphology and temperature of these complexes. To conclude this section on protostars, the value of using UKT14 with its associated polarimeter [33] to detect the polarization properties of protostars was demonstrated by Holland *et al.* [34] who observed the polarized 800 μm emission from aligned dust grains in the prototypical Class 0 source VLA 1623 and in Sharples 106-IR, a high-mass YSO along with its associated protostar S 106-FIR. For VLA 1623, the magnetic field was found to be almost exactly perpendicular to the highly collimated CO outflow, suggesting that the outflow is not collimated by the magnetic field. However, for the S 106 region, the situation was more complex, and it was clear that more extensive imaging would be the way forward, and that would have to wait for SCUBA.

### 2.3. Stars and discs

The study of nearby stars and their associated circumstellar discs attracted a lesser degree of attention, probably because most of the observations turned out to be very difficult and at the limit of the capability of UKT14. On the other hand, the observations provided unique insights into a number of phenomena. Mannings & Emerson [35] observed six T Tauri stars to investigate the dusty, circumstellar discs surrounding these stars in the early stages of stellar evolution using UKT14’s full filter set of 2 mm to 350 μm. For the optically thin sources, the spectral indexes indicated that the dust grains were larger than found in the interstellar medium, suggesting that grain growth in the protoplanetary discs had already occurred and was ongoing. The authors estimated that the rate of growth was of order 10^6^ M_⊙_ per year.

The Vega phenomenon (excess thermal emission above that expected from the stellar photosphere at far-IR wavelengths) was first discovered by the *IRAS* satellite and subsequently investigated by several UKT14 observational campaigns. The breakthrough was made by Zuckerman & Becklin [36] who made the first detections of excess 800 μm emission from the stars Vega, *β* Pictoris and Fomalhaut. Sylvester *et al*. [37] followed up with observations from 2 mm to 450 μm in a major paper describing the far-IR emission from a large sample of stars including Vega. Nine stars were detected at millimetre/submillimetre wavelengths and the data suggested that the surrounding dust was generally more likely to be in a ring rather than a spherical cloud and that they were composed of larger grains than found in the interstellar medium (ISM). Although there remained some uncertainty about the precise evolutionary state of all of the stars (some may have been younger than the main sequence), nevertheless, these important papers pointed the way forwards to some of the earliest and most important observations that SCUBA would make (see §3.4).

The extended dusty envelope of five highly evolved stars (including one planetary nebula) were observed by Knapp *et al.* [38] who found that the spectral index was just less than unity for all the sources, irrespective of whether the envelope of the star was carbon- or oxygen-rich. The gas-to-dust ratio was calculated to be around 100. Observations of one of the sources (CRL618) suggested that it might be slowly variable, which was important as one of the primary aims of this programme was to investigate whether these sources might be suitable as calibrators in the millimetre/submillimetre region. Finally, addressing the latest stages of stellar evolution Williams *et al*. [39] used UKT14 as part of a multi-frequency study of the Wolf–Rayet system WR 147 and showed that the presence of non-thermal emission between the two stars was most probably due to colliding winds in the system.

### 2.4. The Galactic Centre

The Galactic Centre region was a difficult target for UKT14 due to the complex emission over an extended region and the southerly declination. Goldsmith *et al.* [40] set the scene with observations of the Sgr B2 molecular cloud at 1100, 850, 450 and 350 μm to give the first detailed submillimetre picture of this complex region. The maps, by themselves a major achievement with the single-pixel UKT14, revealed a number of individual sources and a variety of optical depths. The two previously known (compact central and northern) sources dominate the emission but two new sources were detected, associated with HII regions/molecular complexes. Dent *et al*. [41] made maps of the 10–20 pc region at 1100 and 800 μm, as well as the inner region at 450 μm. The 2 pc inner ring was clearly detected, as was the dust emission from three giant molecular clouds, which seemed to be connected by a ridge of thermal emission. The 2 pc ring revealed a two-component structure in the submillimetre region: northern and southern emission, which were bounded by the radio continuum spiral arms. Zylka *et al.* [42] then made maps at 800, 600 and 450 μm of the 2 arcmin region surrounding the strong radio source Sgr A*. This was one of the rare examples of the difficult-to-calibrate 600 μm data being used from UKT14, especially in a map. A number of important conclusions were derived from these observations: that warm dust emission was definitely responsible for most of the far-IR emission from the region and that the heating of the dust was not from the central supermassive black hole but from a cluster of hot and luminous stars in the central parsec region.

### 2.5. Dust emission in external galaxies

Although the *IRAS* satellite had detected strong dust emission from many relatively nearby galaxies, the low surface brightness and the small size of the JCMT beam in comparison made UKT14 detections relatively difficult. Chini *et al.* [43] carried out observations at 800 and 400 μm of seven of the 32 spiral galaxies that were previously mapped at 1.3 mm by the IRAM 30 m telescope. It was found that the sample split into two, with one-half being dominated by cold interstellar dust with a temperature of approximately 20 K, while for the rest, much colder dust (approx. 10 K) dominated. The ratio of the infrared luminosity to the gas mass turned out to be equivalent to the star formation rate in the Milky Way. Fich & Hodge [44] observed a sample of 22 early-type galaxies detected by *IRAS* and managed to detect six of them, with tight upper limits being obtained on a further eight. These allowed upper limits to be determined for the dust temperatures, the upper limit being principally because of the very extended size of the *IRAS* beam compared to that of UKT14 on the JCMT. Depending on the value of the emissivity, these temperatures lay between 20 and 40 K.

**Figure 3.**
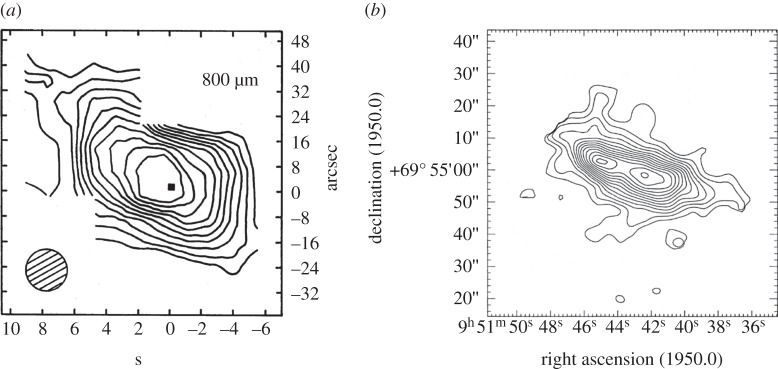
UKT14 imaging of the starburst galaxy M82 at 800 μm (*a*) and at 450 μm (*b*). The higher angular resolution afforded by the 450 μm observations revealed the double-peaked central structure for the first time at submillimetre wavelengths. Adapted from Hughes *et al.* [45,46].

Mapping of nearby galaxies was a difficult proposition, requiring the most stable and driest conditions and so the results were relatively sparse given the fickle nature of matching observing conditions to requirements with the fixed-date scheduling at the time. However, two programmes stand out. Hughes *et al*. [45] followed up their earlier maps of the nearby starburst galaxy M82 at 1100 and 800 μm by succeeding in making a diffraction-limited map of the 1.5 kpc diameter nuclear regions at 450 μm [46]. The 9 arcsecs resolution of the map showed that the thermal emission from the central dust cloud was double in structure and the dust temperature was around 48 K. Both the earlier 800 μm map and the 450 μm image are shown in figure 3. Hawarden *et al*. [47] made an 800 μm map of the central region of the nearby radio galaxy NGC 5128 (Centaurus A); a difficult observation due to the very southerly declination of the source. The map was combined with photometry at all the UKT14 filters apart from 600 μm and the results showed that the non-thermal central emission was surrounded by a circumnuclear torus of dust. Farther out from the centre, extended dust emission was observed and even farther out, the dark optical dust lanes in the galaxy were also detected. Further advancements were not made until SCUBA was able to map the extended cold dust reservoirs in a number of nearby spiral galaxies (see §3.2).

Many programmes sought to detect submillimetre thermal emission from dust in radio-quiet quasars and active galactic nuclei (AGN), and in 1992, Barvainis *et al.* [48] made the breakthrough by detecting the Seyfert I galaxy PG1434+590 and the gravitationally lensed quasar 1413+117 (the ‘Cloverleaf’) at 450 and 350 μm; the latter was also detected at 800 μm. The measured spectral indexes favoured thermal emission from dust, but non-thermal, synchrotron emission could not be completely ruled out. At a redshift of 2.546, the strong suggestion was that this was the emission from typical far-IR galaxies red-shifted into the submillimetre. This was a milestone observation. At the same time, Clements *et al.* [49] detected the high-redshift (*z*=2.286) *IRAS* galaxy 10 214+4724 at 800 and 450 μm. The authors concluded that the dust-enshrouded Seyfert model and the primeval galaxy model were both excluded by their observations but the submillimetre emission was consistent with a massive starburst of around 100 M_⊙_ per year. However, the observations were unable to determine whether this particular starburst was responsible for the formation of a significant fraction of the stars in the galaxy and, indeed, much of the dust probably existed from previous bursts of star formation. This work was immediately followed up by Hughes *et al*. [50] who detected three out of a sample of 10 *IRAS*-selected radio-quiet quasars at 800 and 450 μm. In this case, the very steep spectral index confirmed that the emission was indeed thermal radiation from warm dust and conclusively ruled out any significant non-thermal synchrotron contribution. The dust grain temperature was estimated to be in the range 45–60 K. The redshift limit was further extended by Isaak *et al*. [51] who, in 1993, observed the quasar BR1202-0725 at a redshift of 4.69, detecting dust emission at 1100 and 800 μm with a best fit temperature of 53 K.

*IRAS* detections provided the sources for many samples of submillimetre observations, and a number of multi-wavelength investigations of ultraluminous *IRAS* galaxies (ULIRGs) were undertaken using UKT14. An example of one of these was by Rigopoulou *et al*. [52] who detected nine out of the 10 brightest ULIRGs at 350, 450, 800 μm and 1.1 mm. One of the key aims of this programme was to determine whether the far-IR emission was powered by thermal emission from dust in a starburst (as in the above), or by accretion onto a central supermassive black hole. The observations showed that, for these galaxies, the submillimetre emission was consistent with thermal emission. Although two of the objects might have housed an AGN, their very weak X-ray emission implied a very large optical depth and hence again argued strongly against accretion being responsible for the far-IR luminosity.

Moving on to radio galaxies, in 1994 Dunlop *et al*. [53] investigated the concept that the stars in elliptical galaxies were believed to have been formed in a rapid bust of star formation in the early Universe. They used UKT14 observations to search for evidence in the form of thermal emission from dust from the anticipated starburst phase in these distant galaxies; this same dust making them very faint for optical studies. From a sample of six high-redshift radio galaxies they were successful in detecting the redshift 3.8 galaxy, 4C41.17, at 800 μm with a strong upper limit at 450 μm. These two points were sufficient to establish thermal emission from dust at a temperature around 40 K and that the dust mass was greater than 10 times that found in corresponding low-redshift radio galaxies. The dust luminosity was approximately 5×10^13^ solar luminosities (L_⊙_), corresponding to a starburst of a few thousand M_⊙_ per year. The authors concluded that the observations were consistent with approximately 10% of the eventual mass of the galaxy still to be converted into stars and that it was possible that we were witnessing the end-product of star formation that had begun at much higher redshifts. However, it was not possible to say more about the formation epoch of elliptical galaxies *per se*. This was probably the earliest UKT14 observation relating to cosmology and the epoch of galaxy formation and early evolution.

These exciting high-redshift studies were continued and the context of unambiguously identifying dust emission from relatively low signal-to-noise observations were discussed in detail by Hughes *et al*. [54], reviewing the observations to date, including some recent and very faint detections at 800 μm from their ongoing programme. This was an important overview, and set the scene for the seminal work that was to follow with SCUBA (see §3.1).

### 2.6. Flat spectrum radio sources and blazars

Extensive programmes of observations of flat-spectrum radio sources, in particular blazars, were a feature of UKT14 on the JCMT, following on from earlier work on UKIRT. These observations sought to answer specific questions for these relativistic-jet dominated sources: to determine the ‘snapshot’ spectral energy distribution from radio to gamma-ray regions; to monitor and determine the variability behaviour; to test theoretical models such as the ‘shock-in-jet’ model of Marscher & Gear [55]. Being bright, these sources were readily observable in many weather conditions and also required only short integration times. They were also a feature of the Discretionary Time available to the Director of the telescope. They featured prominently in many coordinated multi-wavelength campaigns from the radio to gamma rays.

Stevens *et al.* [56] published the last in the series of multi-frequency observations of blazars in 1994 which covered 17 blazars at wavelengths from 800 μm to 13 mm. Good agreement was found between the variability and the shock-in-jet models and it was found that the flares in the BL Lac objects tended to reach a maximum at a longer wavelength than those of the optically violently variable (OVV) quasars. This might indicate a stronger shock in the former objects. The data also showed that the flaring behaviour was complex, with multiple maxima and flickering being present and that ‘clean’ flares were relatively rare, all of which indicated greater temporal sampling being needed.

Gear *et al*. [57] further investigated the differences between the BL Lacs and OVVs through a large sample of 22 of the former and 24 of the latter. Quasi-simultaneous data were obtained across a wide wavelength range, which showed that the overall spectral shape was relatively consistent across all the sources. This indicated that the same basic mechanism was at work in both classes; however, as noted above, a clear difference was again found in the millimetre-region spectra, indicating a subtle difference in the jet properties between the two classes of flat-spectrum radio source. The authors suggested that the parent sources for the two classes might be the Fanaroff–Riley Class I and Class II sources, in which case submillimetre polarimetry would be an acid test for the future.

This was eventually undertaken using the UKT14 polarimeter to observe 26 flat-spectrum radio sources at 1.1 mm and 800 μm [58]. Although a significant level of linear polarization was detected in most sources (of order 10–15%), the magnetic field seemed less well ordered on sub-parsec scales than on parsec scales and in the most highly ordered cases it was perpendicular to the jet axis. No significant difference was found between the BL Lac and the flat-spectrum quasars and while the emission from many of the most highly polarized sources could be well fitted by shock-in-jet models, for most sources this was not the case. Conical shock models seemed to be the best descriptor for the diverse emission from the jets in the sample.

Extensive multi-frequency observing campaigns from radio to gamma rays were carried out on particular sources, for example, results from the quasar 3C279 were reported by Hartman *et al*. between 1996 and 2001 [59,60]. These extensive observations showed that the variability was very complex, with different correlations being seen for different flares. The spectra could be best modelled with a relativistic electron-dominated jet with gamma-ray production arising through a combination of synchrotron self-Compton and external Compton processes. Interestingly, when 3C279 was in its high state, the gamma-ray luminosity dominated everything else by at least a factor of 10.

Another very popular quasar for multi-frequency observations was 3C273, typified by the work by Robson *et al.* [61] who reported on a 4-year observing campaign from infrared through centimetre wavelengths during which a number of flares were seen. A period of relative inactivity allowed the quiescent spectrum to be obtained, which could then be subtracted from the flaring behaviour to determine the flare emission itself. Caution was noted in that the behaviour of a flare was critically dependent on the temporal overlap of the observations at differing wavelengths, but where these were simultaneous, the infrared emission preceded that at longer wavelengths and there was distinct evidence for the evolution of the turnover of the flare to propagate to longer wavelengths. The emission between the infrared and 2 mm wavelengths was commensurate with a single synchrotron component associated with the innermost part of the relativistic jet or the injection zone itself. A major paper by Turler *et al*. in 1999 [62] reported on 30 years of multi-wavelength monitoring of 3C273 showing the complex behaviour of this source and providing a database for emission modelling purposes.

With the demise of UKT14 and the introduction of SCUBA, these programmes tended to fall in popularity due to the high impact of imaging science compared to single-pixel photometry. However, the work exploring dust emission from radio-quiet galaxies and AGNs at cosmological redshifts was ideally suited to SCUBA, as will be seen in the next section.

## 3. The first camera arrays: the SCUBA era

SCUBA, the Submillimetre Common-User Bolometer Array, built by the Royal Observatory Edinburgh, was in the late 1990s the most versatile and powerful of a new generation of submillimetre cameras [3]. It uniquely combined a sensitive dual-waveband imaging array with a three-band photometer, and had a sensitivity background-limited by the emission from the Mauna Kea atmosphere and telescope at all observing wavelengths from 350 μm to 2 mm. The increased sensitivity and array size meant that SCUBA mapped approximately 10 000 times faster than UKT14 to the same signal-to-noise ratio. Most importantly, SCUBA was a facility instrument, open to the world community of users, and was provided with an unprecedented high level of user support.

The dual-camera system consisted of a short-wavelength (SW) array of 91 pixels and a long-wavelength (LW) array of 37 pixels. Each array had approximately the same field-of-view on the sky (2.3 arcmin in diameter) and could be used simultaneously by means of a dichroic beamsplitter. The SW array was optimized for operation at 450 μm (but could also be used at 350 μm), while the LW array was optimized for 850 μm (with observations at 750 and 600 μm also possible). The array pixels were arranged in a close-packed hexagon, with the photometric pixels positioned around the outside of the LW array. The detectors were cooled to approximately 100 mK using a dilution refrigerator, ensuring close to a factor of 10 increase in sensitivity per pixel over UKT14. SCUBA was delivered to the JCMT in April 1996, and first light on the telescope was obtained in July. After extensive commissioning, the first astronomical data for the community were taken in May 1997 using two modes of operation: photometry and ‘jiggle-mapping’, the latter using novel movement of the secondary mirror to create a fully sampled image. The final major mode of data acquisition, ‘scan-mapping’, was released in February 1998. Although issues with the filter drum meant that SCUBA became a fixed 450/850 μm imager by the early 2000s, the popularity of the instrument remained very high during its entire lifetime.

### 3.1. New perspectives on galaxy formation and evolution

In late 1997, SCUBA made several monumental discoveries, particularly in the area of galaxy formation and evolution. Capitalizing on a spectacular period of good weather on Mauna Kea (the winter El Niño event of late 1997/early 1998), observations revealed a population of galaxies responsible for at least part of the far-IR background, detected a number of high-redshift galaxies and provided new insights into galaxy evolution. In the next section, we summarize just a few of these high-profile discoveries.

#### 3.1.1. ‘SCUBA galaxies’

The most vigorously star-forming galaxies in the nearby Universe are also those in which dust obscuration is the most significant. It was long suspected, therefore, that the early evolution of galaxies would take place inside shrouds of dust. The first deep SCUBA maps outside of the Galactic Plane immediately confirmed this suspicion, revealing a large population of hitherto unknown, star-forming galaxies. This discovery was reported by Smail *et al.* [63] in a series of targeted observations towards lensed galaxy clusters, exploiting the amplification of all background sources by the clusters. The authors concluded that the observed source counts needed a significant increase in the number density of star-forming galaxies in the high-redshift Universe and suggest that previous optical surveys may have underestimated the star formation density by a large factor. This work was the first peer-reviewed paper to emerge from SCUBA observations.

**Figure 4.**
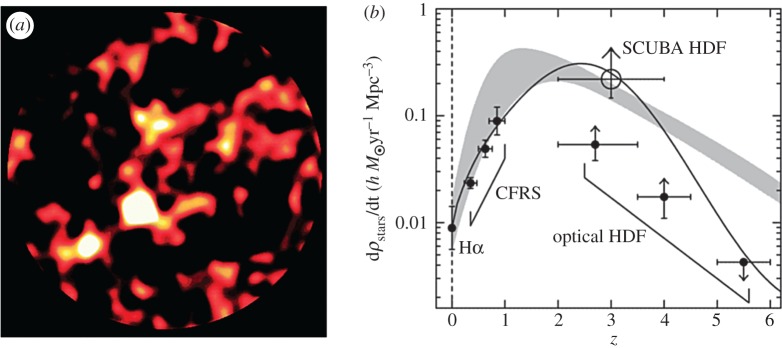
(*a*) The SCUBA 850 μm image of the Hubble Deep Field. (*b*) The global star-formation history of the Universe. The mean star formation rate in the Universe is plotted against redshift, implying a rate a factor of 5 higher based on the SCUBA observations compared to values obtained from previous optical measurements [65]. Adapted from Hughes *et al.* [65].

Subsequent unbiased (blank-field) surveys by groups led by Barger [64], Hughes [65] and Eales [66] confirmed that the surface density of submillimetre sources was several orders of magnitude above that expected for a non-evolving galaxy population. The conclusion was that strongly star-forming galaxies must have been substantially more common in the early Universe than they are today. Having an instrument with hitherto unprecedented imaging capability and sensitivity meant that SCUBA could maximize the use of good-weather periods for statistically significant wide and deep surveys. For example, 14 nights of some of the best weather seen on Mauna Kea^1^ were used to produce the deepest ever submillimetre image to date. The 850 μm image of the Hubble Deep Field (HDF) by Hughes *et al*. [65] reached a 1*σ* noise limit of 0.7 mJy beam^−1^ at 850 μm over an area of around 5 arcmin^2^. It was concluded that the radiation from the five most significant detections in this iconic image, as shown in figure 4*a*, accounted for 30–50% of the previously unresolved background emission in the HDF area. The star formation rate implied from these redshift 2–4 galaxies was a factor of 5 higher than that inferred from optical observations (figure 4*b*). The paper describing this seminal discovery has at the time of writing (June 2017) reached over 1000 citations.

Blain *et al.* [67] went on to conclude that these first deep submillimetre surveys confirmed that a large population of dusty galaxies was missing from optical inventories of star formation activity. Further support for this was obtained with the submillimetre detection of an extremely red galaxy, HR10, at *z*=1.4 by Cimatti *et al*. [68] and the radio galaxy 8C1435+635 at *z*=4.25 by Ivison *et al.* [69]. The former is a relatively common class of galaxy previously thought to consist of very old, quiescent ellipticals, but which SCUBA revealed to comprise young, star-forming systems similar to local ultraluminous *IRAS* galaxies [68,70]. The distant, submillimetre-selected galaxies discovered by Smail *et al.* [63] were also shown to resemble ULIRGs, based on their rest-frame ultraviolet/optical spectra and a similar proportion of mergers [71,72]. The diversity of SCUBA-selected galaxies was first shown from observations of the distant, galaxies detected in the field of the massive cluster lens Abell 1835 by Ivison *et al.* [73]. One galaxy showed almost pure starburst characteristics, while the others had varying degrees of AGN activity. The study showed that although almost identical spectral energy distributions are seen for many galaxies, they often exhibit strikingly different optical/UV spectral characteristics. It was concluded that optical/UV spectral classifications can hence be misleading when applied to distant, highly obscured galaxies, and that other means of determining the various contributions to the overall energy budget of submillimetre galaxies (and hence to the far-IR extragalactic background) are needed.

#### 3.1.2. Extragalactic surveys go deeper and wider

By the early 2000s, deep extragalactic surveys had become more ambitious and included the 3 and 10 h fields of the Canada–UK Deep Submillimetre Survey [66,74], the 8 mJy survey of the ELAIS N2 and Lockman Hole E fields [75] and wider map of the HDF north region [76]. In this latter work, Borys and co-workers mapped 165 arcmin^2^ of the region surrounding the HDF detecting 19 sources at greater than 4*σ* significance, and concluded that the number of galaxies detected accounted for approximately 40% of the 850 μm submillimetre background. Moreover, the nature of the galaxies uncovered in these surveys was becoming clearer, with critical measurements such as the determination of a median redshift of 2.4 from radio measurements reported by Chapman *et al.* [77].

**Figure 5.**
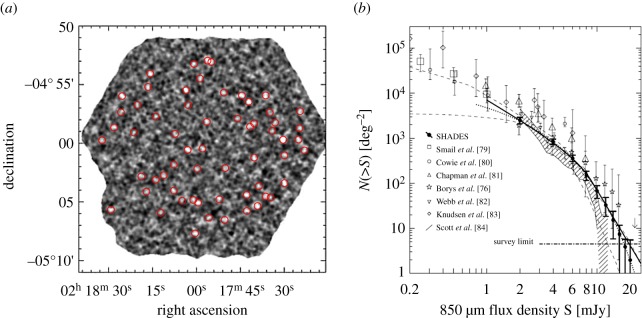
(*a*) The 850 μm SCUBA image of the 406 arcmin^2^ SXDF/UDS SHADES field, identifying some 60 sources. (*b*) Cumulative combined SHADES source counts compared to previous estimates. Adapted from Coppin *et al.* [78].

Towards the end of 2002, the first data were taken in what was to be the most ambitious extragalactic survey undertaken to date at the JCMT. This major, collaborative survey was called SHADES (the SCUBA HAlf Degree Extra-galactic Survey) and aimed to cover 0.5 degree^2^ to a 4*σ* detection limit of 8 mJy beam^−1^ at 850 μm. SHADES was motivated by many science drivers, particularly the desire to clarify the number density, redshift distribution and clustering properties of the bright submillimetre-selected galaxy population. To make further progress in this field required a large and complete sample of 850 μm sources (analogous to the 3C radio source sample, which ultimately revolutionized extragalactic radio astronomy). The main issue (particularly for the non-cosmologists that used the JCMT!) was that the survey would require approximately one-third of the usable UK time on the telescope over the subsequent 3 years. The resulting 850 μm maps of the Lockman Hole and SXDF/UDS fields (the latter is shown in figure 5*a*) formed the largest submillimetre imaging survey of meaningful depth ever undertaken to date, and provided a uniquely powerful resource for the study of the bright submillimetre galaxy population. The results from this survey, reported by Coppin *et al*. [78], included a new sample of 120 sources and a definitive measurement of the source number counts in the 1–10 mJy range (as shown in figure 5*b*), resolving some of 20–30% of the far-IR background.

In the period between the retirement of SCUBA in 2005 and SCUBA-2 becoming fully operational in 2011, wide-field extragalactic surveys continued on the JCMT using visiting instrumentation. Most notably, Scott *et al*. [85] used the AzTEC continuum camera to survey and construct a source catalogue of 0.15 degree^2^ of the COSMOS field at 1.1 mm. A total of 50 millimetre-wave galaxies were identified with far-IR luminosities typically greater than 6×10^12^ L_⊙_ and implied star formation rates of greater than 1100 M_⊙_ yr^−1^. The survey was centred on a prominent large-scale structure including a massive galaxy cluster at *z*=0.73. In the second paper on the results from this field, Austermann *et al.* [86] reported that the overdensity of submillimetre/millimetre galaxies positionally correlated with individual galaxies seen in the optical/IR studies and also with more tenuous structures in the survey region. The results provided some of the first evidence of the importance of cosmic variance and large-scale structure in the study of submillimetre galaxies (SMGs), and was influential in the design of future surveys with SCUBA-2 (see §4.1).

#### 3.1.3. New insights on radio galaxies and active galactic nuclei

The study of galaxies with AGN was also revolutionized by SCUBA. Early use of the jiggle-mapping mode led to the discovery of SMM02399-0136 by Ivison *et al.* [87], a hyper-luminous galaxy at *z*∼2.8 hosting an AGN. Such galaxies could not be easily detected in conventional AGN/QSO surveys, so the presence of SMM02399-0136 in the very first submillimetre image of the distant Universe suggested that estimates of the prevalence of AGN may require substantial revision. The unprecedented sensitivity of SCUBA’s photometry mode allowed the study of radio-selected and optically selected AGN to move from the pioneering world of bare detections to the reliable extraction of physical parameters. For the high-redshift radio galaxy 8C 1435+635, Ivison *et al.* [69] presented 450 and 850 μm detections of sufficient quality to infer that the formative starbursts of such massive ellipticals may still be in progress at *z*∼4. Observations of a sample of radio galaxies by Archibald *et al*. [88], spanning a range of redshifts between 1 and 5, showed that the submillimetre luminosity of radio galaxies is primarily a function of redshift, and furthermore may be representative of massive ellipticals in general. The authors concluded that the observed increase in the submillimetre detection rate and characteristic luminosity with redshift is due to the increasing youthfulness of the stellar population of radio galaxies in their sample.

The steep-spectrum, narrow-line radio galaxy 53W002 was especially interesting as it had been shown to contain an overdensity of compact, Ly-*α* emission-line galaxies at *z*∼2.4. SCUBA observations of the 53W002 field by Smail *et al.* [89] uncovered four luminous submillimetre galaxies. By matching the submillimetre source position using an astrometrically precise 1.4 GHz map, one of these sources was shown to be coincident with a Lyman-*α*-selected galaxy at *z*=2.39, 330 kpc away from the radio galaxy in projection. This confirmed the presence of ultraluminous, dusty galaxies in the overdense structure around 53W002 at a look-back time of approximately 11 Gyr. SCUBA galaxies, as the progenitors of massive elliptical galaxies, should therefore be strongly clustered in the highest density regions of the distant Universe.

#### 3.1.4. Gamma-ray bursts: galaxy evolution at high redshifts

An alternative method for studying the characteristics and evolution of galaxies at high redshift is to use gamma-ray bursts (GRBs). SCUBA pioneered early observations both of the host galaxy and of the afterglow from GRBs, speculating that the hosts were early starburst galaxies in contrast with the previously SCUBA-selected galaxies, which tended to host populations of more evolved stars. The implication was that the submillimetre surveys, which have certain selection biases, miss a fraction of the cosmic star formation, which can be possibly recovered by observations of GRB hosts. Although the observations were somewhat of a struggle (due to low flux levels), and only a few host galaxies were detected in the submillimetre region [90], the results paved the way for future studies, particularly from space missions such as *SWIFT*, *Spitzer* and *Herschel*. For some bursts the early afterglow (hours to weeks following the burst itself) peaks in emission in the submillimetre region. By tracking the evolving afterglow emission across the entire spectrum, Smith *et al*. [91] showed that it was possible to study aspects such as the types of shocks involved, whether the outflow has a jet or spherical geometry, and to also investigate the geometry of the surrounding medium (uniform versus prior stellar wind).

#### 3.1.5. Probing large-scale structure: the Sunyaev–Zel’dovich effect

One of the most versatile probes of large-scale structure of the Universe is the Sunyaev–Zel’dovich (SZ) effect—the distortion of the cosmic microwave background (CMB) radiation through inverse Compton scattering by high-energy electrons in galaxy clusters. This distortion produces a characteristic ‘increment’ in the CMB temperature above frequencies of around 200 GHz, an effect that had only been measured in eight clusters before SCUBA made observations of a further two compact galaxy clusters, thereby providing a significant addition to this field of study. Constraining the full spectral shape of a cluster’s SZ distortion allows separation of the thermal SZ effect, which is caused by the random motions of the cluster’s electrons, from the kinetic effect, caused by the cluster’s motion relative to the CMB rest frame. For the observations a large (180 arcsecs) chop throw of the secondary mirror had to be employed to ensure that no significant SZ flux (a small-amplitude signal) appeared in the reference beams. In addition, 450 μm data were used to remove the effects of atmospheric emission from the 850 μm data because standard, in-band atmospheric corrections would cancel the SZ intensity. The JCMT’s high angular resolution also allowed rejection of possible point-source contaminants which plague SZ measurements with smaller aperture instruments. The results of this work by Zemcov *et al*. [92] provided robust, high S/N measurements of the SZ increment towards the clusters Cl 0016+16 and MS 1054.4−0321.

### 3.2. The nearby Universe: cold dust and giant magnetic bubbles

**Figure 6.**
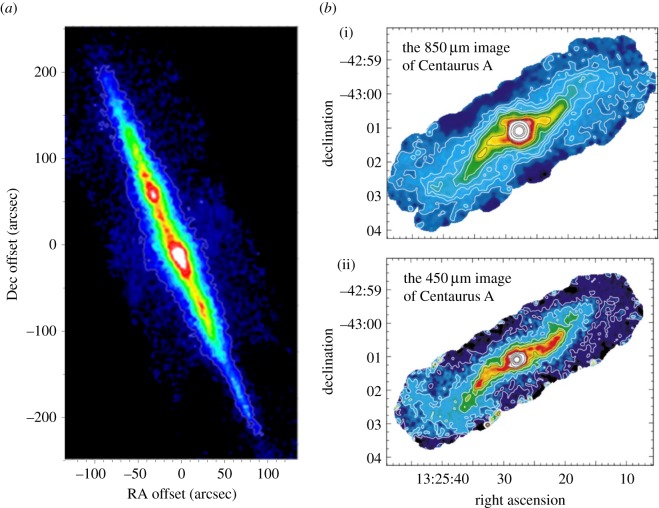
(*a*) SCUBA 450 μm image of NGC 891 highlighting regions of cold dust extending to large galactic radii; (*b*) 850 μm (i) and 450 μm (ii) SCUBA mosaic image of the nearest giant elliptical galaxy Centaurus A, showing a warped ‘S-shaped’ inner disc. The NGC 891 figure is adapted from Alton *et al.* [93], and Centaurus A from Leeuw *et al.* [94].

Another key area of research focused on using SCUBA’s sensitivity and mapping capabilities to make the deepest images to date of the location of cold dust reservoirs in nearby spiral galaxies. The bulk of star formation activity in nearby spirals is often missed by IR studies, because most of the dust mass resides in cold, extended, low-surface-brightness discs, often far from the galactic nucleus. Studies of nearby galaxies such as NGC 891 (figure 6*a*) and NGC 7331 by Alton *et al*. [93,95] revealed that up to 90% of the total dust mass can be located within galactic discs at large radii. The images also detected spectacular dust ‘chimneys’ escaping from the main absorption layer up to *z*-heights of nearly 2 kpc. Further observations of cold dust emission in the ‘Whirlpool Galaxy’ (M51) by Meijerink *et al.* [96] showed that the 850 μm originated in an underlying exponential disc with a scale length of 5.5 kpc. This reinforced the view that the submillimetre emission from spiral galaxy discs traces the total hydrogen column density (i.e. the sum of H_2_ and HI).

Dunne *et al*. [97] observed 104 galaxies from the *IRAS* Bright Galaxies sample to provide the first statistical survey of the submillimetre properties of the local Universe. They made the first direct measurements of the submillimetre luminosity function, concluding that the slope of the function must flatten at luminosity values lower than in the survey. They postulated the existence of a population of ‘cold’ galaxies (less than 25 K) emitting strongly in the submillimetre region that would have been missed from far-IR selected samples. By comparing the global galaxy properties with their submillimetre/far-IR properties, average gas-to-dust ratios of close to 600 were found, compared to the Galactic value of only 160. The conclusion was that most galaxies in the sample must contain a ‘cold dust’ component with a temperature of less than 20 K.

The sensitivity of SCUBA was also used in earnest to make deep submillimetre images of the central 8×2 kpc region of Centaurus A, the nearest giant elliptical galaxy. The remarkable images at 450 and 850 μm by Leeuw *et al.* [94] revealed an unresolved central AGN core, an inner jet interacting with gas in a dust lane, an S-shaped inner disc of active star formation and a colder outer disc. These images are shown in figure 6*b*. The results supported the theory that the inner disc material is consistent with a warped-disc model of tilted rings. Finally, with magnetic fields believed to play an important role in the star formation process in the central region of starburst galaxies, Greaves *et al.* [98] used the SCUBA polarimeter [99] to map the magnetic field morphology surrounding the inner regions of M82. The polarized dust emission showed that the major magnetic features found in M82 are ordered fields over scales of hundreds of parsecs within the torus, and an outer ‘bubble-like’ field associated with the dusty halo, with a diameter of at least 1 kpc.

### 3.3. Large-scale mapping of the Galactic Centre

**Figure 7.**
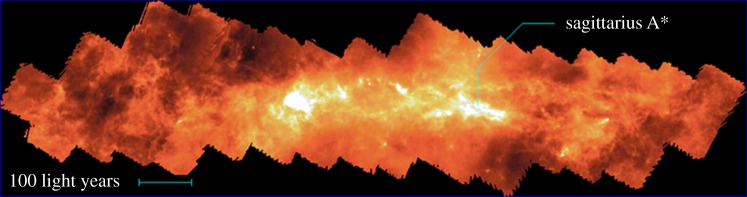
The SCUBA 850 μm image of CMZ of the Galactic Centre, covering a region of 3×0.6° including the cloud populations near Sgr A*. Adapted from Pierce-Price *et al.* [100].

As described in §2.4, mapping large areas, such as the Galactic Centre, was painfully slow and difficult with single-pixel photometers. One of the most ambitious, large-scale projects undertaken with SCUBA was to map the Central Molecular Zone (CMZ) of the Galactic Centre over an extent of 3° in galactic longitude. The final image, a mosaic of over 50 individual fields, by Pierce-Price *et al*. [100], shown in figure 7, contains detailed information on both the warm cloud population near Sgr A* (the non-thermal radio source at the centre of the Galaxy), and the circumnuclear disc, with a 1*σ* sensitivity limit of approximately 30 mJy beam^−1^ at 850 μm, equivalent to just a few M_⊙_. There is clearly an extraordinary amount of structure, and such data are vital for understanding cloud evolution in a dynamic (sheared and rapidly rotating) environment. The images also provide input to models of the starburst phenomenon in other galaxies as well as the periodic star formation inferred in our own Galactic Centre. The SCUBA data represented the first optically thin map to trace essentially all the mass in the CMZ at high spatial resolution.

Aitken *et al*. [101] reported the first detections of linear polarization from Sgr A* at 750 μm, 850 μm, 1.35 mm and 2 mm, confirming the contribution of synchrotron radiation. Large changes in the position angle between the submillimetre and millimetre measurements were observed and the best model to explain these changes was one in which the synchrotron radiation from the excess flux is self-absorbed in the millimetre but becomes optically thin in the submillimetre region. The authors conclude that this suggests the flux originates from an extremely compact source of approximately 2 Schwarzschild radii.

### 3.4. Debris discs: the fallout of planetesimal collisions around stars

**Figure 8.**
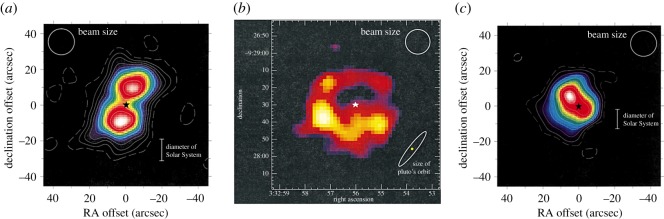
A selection of 850 μm images of debris discs observed using SCUBA: (*a*) Fomalhaut, (*b*) *ϵ* Eridani and (*c*) Vega. The projected diameter of the Solar System (size of Pluto’s orbit) at the distance of each star is shown on each image. Adapted from Holland *et al.* [102] (Fomalhaut and Vega) and Greaves *et al.* [103] (*ϵ* Eridani).

As described in §2.3 observations with UKT14 had already shown an intriguing glimpse (via point-by-point photometry) of what was possible in terms of imaging the faint discs that surround many main sequence stars. The fact that such material exists suggests the presence of larger unobservable bodies in these systems, such as planets. SCUBA was well suited to measure the low-level thermal emission from the dust grains in such discs. The results from the work of Holland *et al*. [102] were spectacular, and included the first images of the debris discs around the well-known stars Fomalhaut and Vega. For example, around Fomalhaut, the peak flux in the map (figure 8*a*) was seen to occur in two distinct regions, offset from the stellar position. The image is consistent with an edge-on torus (doughnut-like) structure of a size similar to our own Edgeworth–Kuiper Belt (EKB), and with a central cavity containing significantly less dust emission. The cavity is about the diameter of Neptune’s orbit, and a possible explanation is that the region has been cleared of gas and dust by the formation of planetesimals [102]. More recent observations by the *Hubble Space Telescope (HST)* [104], *Herschel* [105] and the Atacama Millimeter Array (ALMA) [106], at higher angular resolution, have shown that the disc is actually a thin ring, possibly shepherded by one or more planets.

How typical is our Solar System architecture around other stars is one of the most fundamental questions in astronomy. Fomalhaut and Vega (figure 8*c*) are extremely luminous, relatively short-lived A-stars, and hence any planetary system that may exist around them would probably be very different from our Solar System. Further work with SCUBA therefore targeted G and K stars in an attempt to address the uniqueness of our Solar System architecture. The image of the debris disc around the young, nearby (only 3 pc) *ϵ* Eridani by Greaves *et al.* [103], shown in figure 8*b*, revealed a dust ring peaking at 60 AU from the star with a void of emission in the inner 3 AU radius [103]. Substructure, observed as asymmetries within the ring, was interpreted as possibly being due to perturbations by planets. Moreover, observations of the Sun-like G8 star *τ* Ceti by Greaves *et al*. [107] revealed a vast EKB-like disc. Modelling showed that the mass in colliding bodies up to 10 km in size is around 1.2 Earth masses, compared with 0.1 Earth masses in the EKB, and hence the evolution around the two stars has been very different. One possibility is that *τ* Ceti has lost fewer comets from the outskirts of the system, compared with the Sun.

### 3.5. Large-scale mapping of star-forming regions

**Figure 9.**
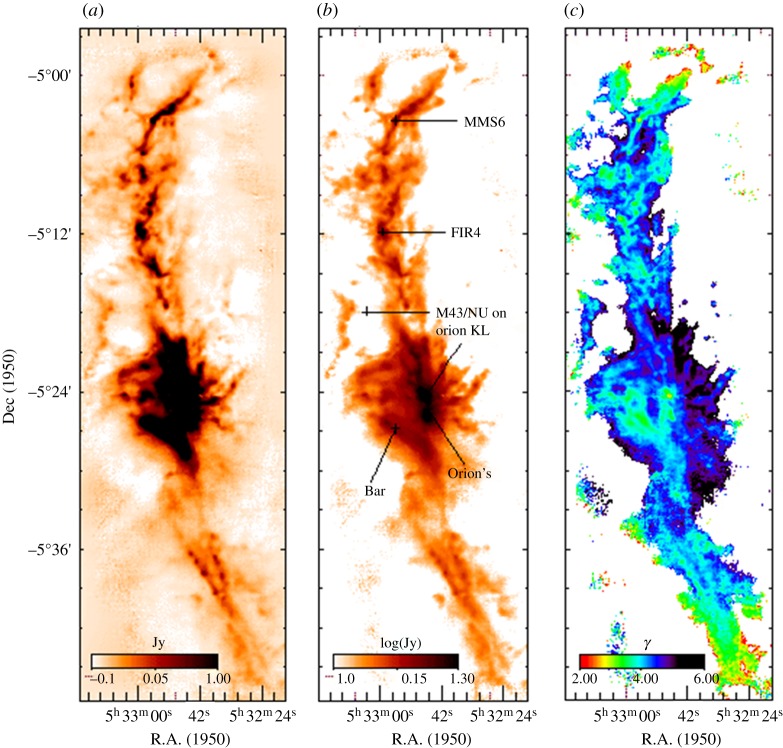
SCUBA image of the Orion A molecular cloud: 850 μm image (*a*) log-intensity image at 850 μm (*b*) and spectral index map (*c*), determined from the ratio of the 450 μm and 850 μm images. The main regions are highlighted in the centre image. Adapted from Johnstone & Bally [108].

One of the key goals for SCUBA was to provide the capability to carry out large-scale (several degrees), high dynamic-range imaging of star-forming regions in the Milky Way. One of the first regions to be imaged was the central region of the Orion A molecular cloud. Figure 9 from Johnstone & Bally [108] shows that the SCUBA images trace the morphology and spectral index of the optically thin emission from interstellar dust. The famous Orion ‘bright bar’ is clearly seen in the image together with a chain of compact sources embedded in a narrow, high column-density filament that extends over the entire length of the map. The region is also believed to be a site of progressive star formation (from the south to the north), and hence offers an opportunity to compare dust core properties (such as the spectral index—figure 9*c*) over a range of evolutionary stages.

Further to the pioneering observations with UKT14 (see §2.2) large-scale mapping of regions such as the *ρ* Ophiuchus molecular cloud by Johnstone *et al.* [109] used clump-finding algorithms to identify, and compare the properties of individual objects. Thus, it became possible to determine the mass distribution of clumps based on submillimetre fluxes for the first time. In the case of *ρ* Ophiuchus, the clumps spanned a mass range of 0.02–6.3 M_⊙_ and the distribution was characterized by a broken power-law, *N*(*M*)∝*M*^−*α*^, where *α* is typically 0.5–1.5. As with other studies, it was concluded that the observed clumps may represent an evolutionary stage, being fragments produced during the collapse of a larger and gravitationally unstable core within the cloud. The observations of the *ρ* Oph A region of the cloud complex also highlighted the vast improvement in performance of SCUBA over UKT14. To achieve the same S/N as SCUBA over the 4×3 arcmin region shown in figure 2 would have taken UKT14 over 10 000 hours!

### 3.6. The Holy Grail: protostars

One of the long-standing challenges facing infrared and submillimetre astronomy is the understanding of the earliest stages of star formation. SCUBA readily demonstrated the power of deep imaging to discover new candidate protostars, as well as obtaining reliable statistics on the early stages of stellar evolution, including the protostellar Class 0 phase. Unbiased surveys of extended dark clouds, for example by Visser *et al*. [110], were also carried out to identify complete samples of protostellar condensations, allowing the measurement of star formation efficiencies, mass accretion rates and evolutionary lifetimes.

Furthermore, as a result of SCUBA observations interest began to focus on the starless (or ‘ pre-stellar’) cores, which are significant in that they constrain the initial conditions of protostellar collapse. Over 40 such cores in the Orion molecular cloud were studied by Nutter & Ward-Thompson [111], who concluded that the high-mass, core mass function (CMF) follows a roughly Salpeter-like slope, just like the initial mass function (IMF) seen in earlier studies. The deep SCUBA maps showed that the CMF turns over at approximately 1.3 M_⊙_, about a factor of 4 higher than the completeness limit. This turnover, never previously observed and only revealed by the much deeper SCUBA maps, mimics the turnover seen in the stellar IMF at approximately 0.1 M_⊙_. The low-mass side of the CMF is a power-law with an exponent of, 0.35–0.2, which is consistent with the low-mass slope of the young cluster IMF of 0.3–0.1. This shows that the CMF continues to mimic the shape of the IMF all the way down to the lower completeness limit of these data at approximately 0.3 M_⊙_.

**Figure 10.**
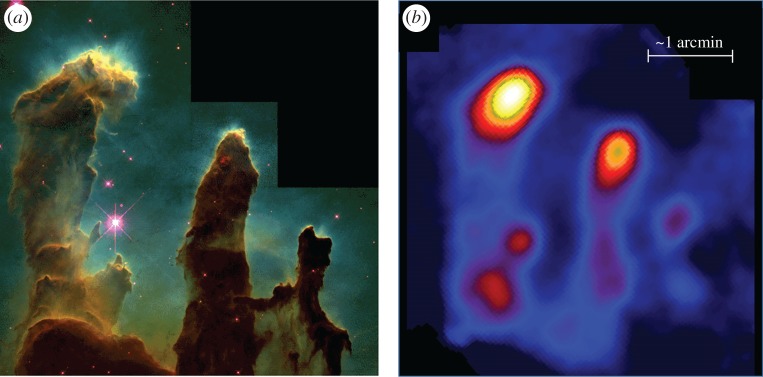
(*a*) The Eagle Nebula (M16) as seen from the *HST* (image courtesy of J. Hester and P. Scowen). (*b*) The SCUBA 450 μm image of the same region highlighting the bright thermal emission from the tips of the ‘fingers’. SCUBA figure adapted from White *et al.* [112]).

One of the the most spectacular images of the earliest stages of star formation came from SCUBA imaging by White *et al*. [112] of the famous Eagle nebula (M16). As shown in the 450 μm SCUBA-2 image presented in figure 10, some differences are immediately evident from the *HST* optical image, particularly in terms of the dominant thermal emission from the tips of the ‘fingers’ seen in the SCUBA map. The continuum spectra of these cores show that they are much cooler (approx. 20 K) than the surrounding molecular gas in each of the fingers. The results of a thermal and chemical model of the environment concluded that the fingers appear to have been formed after the primordial dense clumps in the original cloud were irradiated by light from its own OB stars. During the subsequent photoevaporative dispersal of the cloud, the clumps shielded material lying behind it, facilitating the formation of the fingers. The absence of embedded IR sources or molecular outflows suggest that the cores at the tips of the fingers have the characteristics of the earliest stages of protostellar formation.

One of the most ambitious projects to be undertaken with SCUBA and its polarimeter was an attempt to make the first observations of the magnetic field geometry in pre-stellar cores. By doing so, such results would test the theoretical ideas about the way in which the field geometry affects the star-formation process. The first published maps by Ward-Thompson *et al.* [113] for three cores revealed smooth, uniform polarization vectors in the plane of the sky, showing no evidence of the type of geometry that might be expected in a magnetically dominated stage of evolution. It was concluded that no current model of magnetically regulated star formation could explain the existing observations.

### 3.7. Solar System science: Comet Hale–Bopp and the subsurface of Pluto

It has been long suspected that large particles may be present in comets sufficient to dominate the total mass of the coma. Believed to be products of agglomerative growth in the proto-solar nebula, these particles are the local analogues of the dust observed in the discs around many young stars. Some of the first evidence for this was published for the Comet Hale–Bopp by Jewitt & Matthews [114] based on SCUBA 850 μm observations. The dust coma surface brightness is well described by a steady-state outflow model, in which the dust density varies with the inverse square distance from the nucleus. Submillimetre observations have proved vital in studying the properties of these large particles; the data provide an estimate of the total mass, the dust mass production rate as a function of heliocentric distance and the size of the particles in comparison with those in circumstellar discs.

The subsurface of Pluto is known to contain a reservoir of frozen volatiles but very little is known about it. Greaves *et al*. [115] used archival light curves of the brightness of Pluto to probe just below the skin depth of the thermal changes over Pluto’s day. With the light curve in the submillimetre differing significantly from those measured in the mid- and far-IR, in a region that is optically dark on the planet’s surface, the suggestion is that the layers a few centimetres below the surface have not undergone any major temperature change. One possibility is that these regions could have a different emissivity, perhaps with a subsurface layer richer in nitrogen or methane ices than the surface. Results from the NASA *New Horizons* probe concluded that the surface composition is surprisingly complex, with the nitrogen-, methane- and water-rich areas creating a puzzle for understanding Pluto’s climate and geologic history.

## 4. SCUBA-2: wide-field imaging in the submillimetre region becomes a reality

Although SCUBA had made so many pioneering discoveries, it was obvious by the turn of the century that an even more sensitive camera was required, specifically to allow wide-field surveys to be undertaken, in line with the planned work by satellites such as *Herschel* in the far-IR. The project became known as SCUBA-2, and involved an international partnership between institutes in the UK, USA and Canada. As was the case with SCUBA, SCUBA-2 had two imaging arrays working simultaneously in the atmospheric windows at 450 and 850 μm, the vast increase in pixel count to over 10 000 meant that SCUBA-2 would map the sky 100–150 times faster than SCUBA to the same signal-to-noise ratio. SCUBA-2 was a major step forwards in technology. It was the first astronomical camera to use superconducting transition-edge sensors in a time-domain multiplexed readout scheme. The instrument itself was also a major challenge, having a liquid cryogen-free dilution refrigerator to cool the detector to less than 100 mK and over 600 kg of optics cooled to less than 4 K. SCUBA-2 was delivered to the JCMT in April 2008 with two engineering sub-arrays (one-quarter of the field-of-view at each wavelength), and eventually began science operations in late 2011 with fully populated, science-grade focal planes. In February 2012, SCUBA-2 began a series of unique legacy surveys for the JCMT community. These surveys took almost 3 years and the results provided complementary data to the shorter-wavelength, shallower, larger area surveys from *Herschel*. The SCUBA-2 surveys have also provided a wealth of information for further study with new facilities such as ALMA, and future possible telescopes such as *SPICA* and ground-based large, single-aperture dishes.

### 4.1. The first-generation surveys with SCUBA-2

The key scientific driver for SCUBA-2 was the ability to carry out large-scale surveys of the submillimetre sky to unprecedented depth. Six first-generation ‘legacy-style’ survey programmes were approved covering a very broad base, ranging from the studies of debris discs around nearby stars to galaxy populations and evolution in the early Universe. The SCUBA-2 element of these surveys was initially approved to run from February 2012 to September 2014, with several benefiting from an extension to February 2015. In the next sections, we briefly describe these surveys and summarize some of the key findings so far.

#### 4.1.1. Galactic Plane Survey

**Figure 11.**
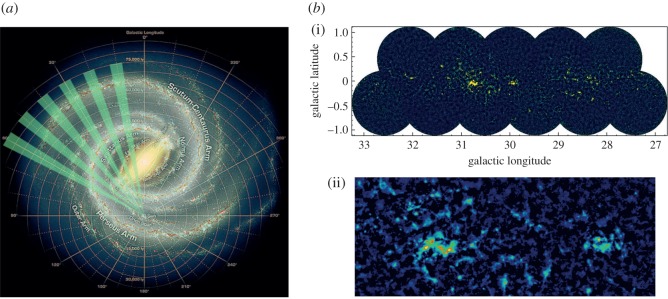
(*a*) The area covered by the JPS (green segments) overlaid on a sketch of the Milky Way by Robert Hurt [116]. (*b*(i)) The *l*=30° full-field image of the Galactic Plane from JPS. (*b*(ii)) Highlighted is the W43 star-forming region from the *l*=30° image. SCUBA-2 adapted from Eden *et al.* [117].

Whereas observations with SCUBA had probed in the inner few degrees of the Galactic Centre (as described in §3.3), the JCMT Plane Survey (JPS) sought to achieve a full census of star-formation activity in the plane of the Galaxy observable from JCMT to a detected mass limit of around 40 M_⊙_ at the far edge of the Galaxy. The aims included examining triggered and large-scale star formation and to study the evolution of massive YSOs, infrared dark clouds and filaments, along with dust evolution and molecular cloud structure. Surveys of the Galactic Plane in the millimetre/submillimetre are currently the only approach to determine the relative importance of the physical processes that are likely to affect the star formation efficiency on Galactic scales (greater than 1 kpc, e.g. spiral density waves) and within individual molecular clouds (e.g. temperature and pressure). To achieve this, the JPS observed six fields along the Galactic Plane at longitudes of 10, 20, 30, 40, 50 and 60° (figure 11*a*), with each field just over 5×1.7° in area as described by Moore *et al.* [116]. The argument was that large fractions of the Plane needed to be surveyed in order to account for the statistical distribution of cloud masses and YSO luminosities, as well as local variance.

The final survey covers an area of approximately 50 degree^2^ and achieved an average noise level of 7.2 mJy beam^−1^ at 850 μm, when smoothed over a beam diameter. An example of one of the fields, highlighting the W43 star-forming region, is shown in figure 11*b*(i)(ii). The survey is approximately 10 times more sensitive than the complementary ATLASGAL survey carried out on the APEX telescope, which studied the inner Galactic Plane at 870 μm, covering galactic longitudes between 60 and 270°. A catalogue of approximately 7800 compact sources was generated from the JPS, and it was shown that these sources are responsible for 42% of the total emission from the maps with the remaining flux lying in filamentary structures. One of the key outcomes of the survey, which also included large-scale observations with HARP (see §5), is that the dominant scale of variations in star formation efficiency in the Galactic disc is that of individual molecular clouds as described in one of the first papers by Eden *et al*. [118], with spiral arms having only a relatively minor influence. Using the *Herschel* 70 μm data, the survey team showed that 38% of the sources detected show evidence of ongoing star formation [117]. The JPS images and associated source catalogue represent a valuable resource for studying the role of environment and spiral arm structure on star formation in the Galaxy.

#### 4.1.2. Gould Belt Survey

The Gould Belt is a large (approx. 1 kpc diameter) ring of molecular clouds and OB star associations that is inclined at approximately 20° to the Galactic Plane. It is important for star-formation studies as it contains most of the nearby low- and intermediate-mass star formation regions such as the Orion and Taurus-Auriga molecular clouds. The inner regions of several of the most prominent clouds had already been studied by SCUBA (as described in §3.5), but it had become clear that wider-scale observations were needed to take in all possible environments and address large-scale structures such as filaments. The JCMT Gould Belt Survey (GBS) aimed to address several of the major unsolved questions in star formation: the evolution of pre- and protostellar cores, the origin of the IMF, and the link between star formation and molecular cloud properties [119]. The targets were molecular clouds within 500 pc of the Sun where the angular resolution is high enough to separate individual pre/protostellar cores (0.1 pc). The survey was awarded 612 hours of observing time, which included both SCUBA-2 and HARP observations of 14 nearby clouds, covering a total area of almost 700 degree^2^. The improved resolution of the JCMT also allows for more detailed study of large-scale filaments, protostellar envelopes, extended cloud structure and morphology down to the Jeans length.

**Figure 12.**
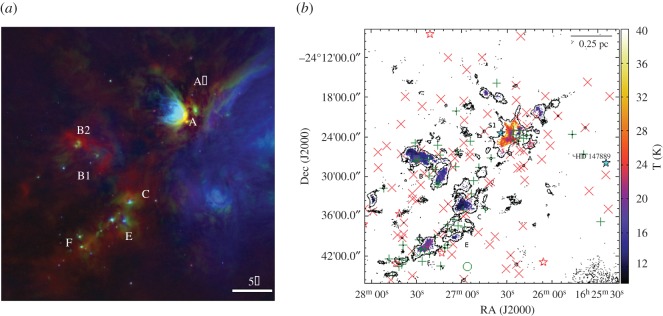
(*a*) Three-colour image of the L1668 region with the main molecular clouds labelled. The red, green and blue channels are from SCUBA-2, *Herschel* 100 μm and *Spitzer* 8 μm, respectively. (*b*) A dust temperature map of the same region determined from SCUBA-2 450 μm and 850 μm flux ratios. Adapted from Pattle *et al.* [120] (L1668 three-colour image) and Rumble [121] (dust temperature map).

The resulting maps of the Gould Belt molecular clouds are among the deepest ever undertaken, with typical noise levels of 3–4 mJy beam^−1^ at 850 μm over tens of square degrees. The uniqueness of the SCUBA-2 observations is that they predominantly trace cold, dense cores that are more likely to be pre-stellar than the more evolved clumps seen by *Herschel*. For example, using observations from both SCUBA-2 and HARP, Pattle *et al.* [120] identified significant fractions of pressure-supported starless cores in Ophiuchus that are unlikely to ever become gravitationally bound. Furthermore, by combining SCUBA-2 data with shorter wavelength data from *Spitzer* and *Herschel*, it is also possible to measure the variation in temperature along the line-of-sight, as shown in figure 12, for the Ophiuchus L1688 cloud complex. Mairs *et al*. [122] presented a catalogue of sources from observations of the Southern Orion A cloud, showing that the larger-scale regions of emission within the cloud are often subdivided into smaller dense fragments that are usually invisible in shorter wavelength surveys. One of the key aims of the survey was to investigate the pre-stellar mass function in the various molecular clouds. Salji *et al*. [123] determined that it peaked at 1.39 M_⊙_ in Orion A, revealing a star-forming efficiency of 14% when compared with the Orion nebula cluster IMF. Furthermore, the pre-stellar mass function was found to decay with a high mass-power-law exponent of 2.53, similar to the Salpeter IMF value of 2.35 for stars in the Solar neighbourhood. The extensive GBS data continues to be analysed and has already produced a number of new source catalogues, characterizing thousands of cores and clumps in terms of their properties and evolutionary status.

#### 4.1.3. Nearby Galaxy Survey

SCUBA pioneered some of the earliest observations of the extent of cold dust in nearby spiral galaxies (see §3.2). As described by Wilson *et al*. [124] the JCMT Nearby Galaxies Legacy Survey (NGLS) aimed to use both SCUBA-2 and HARP to investigate both the physical properties of gas and dust in galaxies, along with the effect that galaxy morphology and unusual environments (such as metallicity) has on the properties of the dense ISM. The SCUBA-2 part of the survey was allocated 100 hours of observing time, with a goal of reaching a sensitivity level of 1.8 mJy beam^−1^ at 850 μm. A total of 48 spiral galaxies were observed, the majority of which came from the *Spitzer* SINGS survey. One of the major findings was the presence of significant levels (up to 25%) of CO in the centres of galaxies. Much of the analysis of the data is still underway; the emission from the majority of the galaxies is very weak, and recovering the flux on scales of several arcminutes has been a challenge for the data reduction. Combining the data with shorter wavelength data from *Herschel* will also allow the dust temperature variation in these galaxies to be mapped across large galactic scales for the first time.

#### 4.1.4. Cosmology Legacy Survey

The JCMT Cosmology Legacy Survey (S2CLS) sought to capitalize on the pioneering high-redshift galaxy work undertaken by SCUBA (as described in §3.1) and other early submillimetre cameras. Submillimetre galaxies (SMGs) are among the most luminous dusty galaxies in the Universe but their true nature remained unclear. It could be that they are the progenitors of the massive elliptical galaxies seen in the local Universe, or a short-lived phase of a more typical star-forming galaxy. As described by Geach *et al.* [125], the key driver of the 850 μm survey was to deliver a sufficient number of galaxies to address this question by reliably measuring the clustering of the submillimetre population (providing valuable constraints on galaxy formation models) and to detect and study the (rare) progenitors of rich clusters. The 850 μm survey would also establish, unambiguously, the faint end of the counts of SMGs in this band. The goal of the deep 450 μm component of the survey was to resolve a significant fraction of the extragalactic background light at 450 μm into individual galaxies by getting as close as possible to the confusion limit at this shorter wavelength (similar to that achieved with SCUBA for the HDF at 850 μm). The survey plan was to map an area of 10 degree^2^ at 850 μm to a depth of 1*σ*=1.5 mJy beam^−1^ and 0.25 degree^2^ at 450 μm to a depth of 1*σ*=1.2 mJy beam^−1^. To achieve this, several extragalactic survey fields (including the original SHADES fields from SCUBA) were to be mapped, for which a wide range of ancillary data is available from other wavelengths. The wide-field 850 μm included a number of well-studied fields, such as the UKIDSS-UDS, COSMOS, *Akari*-Northern ecliptic pole and Lockman Hole north regions, while the ultra-deep 450 μm maps were centred in the COSMOS and UDS fields. The S2CLS was by far the largest of the first-generation legacy surveys, and was awarded close to 1800 hours or 51% of the total survey time over the 3-year period.

**Figure 13.**
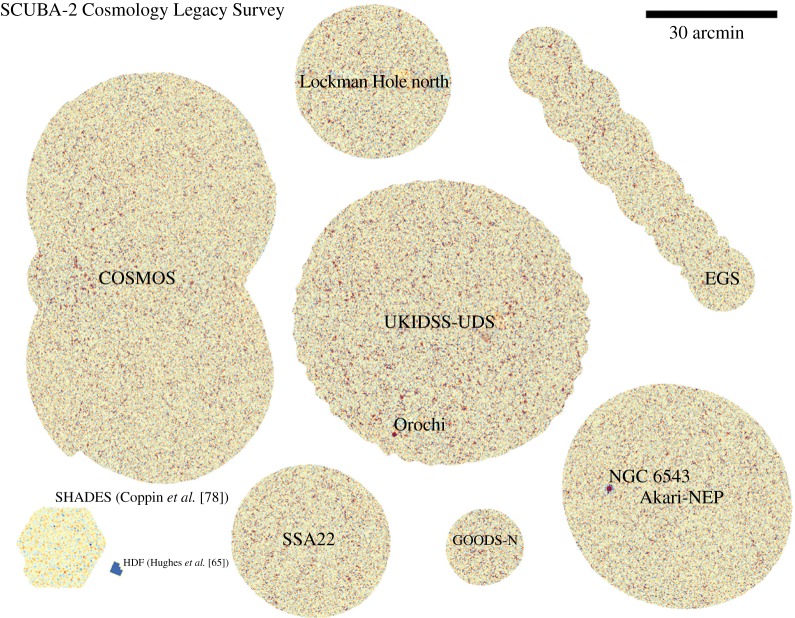
A montage of the 850 μm signal-to-noise maps from the seven extragalactic fields of the SCUBA-2 Cosmology Legacy Survey. For comparison, the previous work on the HDF and SHADES, undertaken with SCUBA, are also shown (see §§i and ii, respectively). Adapted from Geach *et al.* [126].

**Figure 14.**
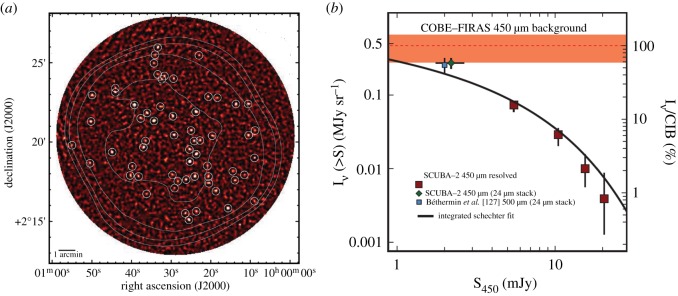
(*a*) SCUBA-2 450 μm signal-to-noise map of the COSMOS/CANDELS field, showing 60 sources detected (circles) at greater than 3.75*σ* significance. The contours show the variation in noise level, starting with 2 mJy beam^−1^ for the inner contour and increasing to 5 mJy beam^−1^ for the outer. (*b*) The integrated surface brightness of the 450 μm sources relative to the cosmic infrared background measured by *COBE*. Adapted from Geach *et al.* [125].

The results from the S2CLS increased the size sample of 850 μm-selected SMGs by an order of magnitude. Figure 13 shows the first 850 μm maps by Geach *et al*. [126] from this extensive survey, covering a total area of approximately 5 degree^2^ and detecting approximately 3000 sources. The average 1*σ* noise in the maps is 1.2 mJy beam^−1^, close to the expected confusion limit of 0.8 mJy beam^−1^. Such a large survey also allows a comprehensive measurement of the number counts of submillimetre sources and the results show both a distinctive upturn in the counts caused by strong gravitational lensing of high-redshift galaxies, and a contribution from local sources of submillimetre emission. For the first unbiased, blank-field assessment of the number counts of galaxies at 450 μm, Geach *et al.* [125] showed that 16% of the cosmic infrared background was resolved into individual galaxies (figure 14), while a further approximately 40% was recovered in the SCUBA-2 map by comparing to *Spitzer*-detected 24 μm emitters. Koprowski *et al.* [128] used multi-frequency data to determine the redshift distribution of the 106 galaxies detected in the deepest, central area of COSMOS field and found a median redshift of 2.38±0.09. Roseboom *et al*. [129] explored the physical properties of these galaxies from their spectral energy distributions, revealing correlations, for example, between the dust temperature and infrared luminosity. Some 24% of the 450 μm sources were found to be starbursts, i.e. displaying an anomalously high star formation rate.

The nature of SMGs was further explored by Wilkinson *et al*. [130] who investigated the clustering of galaxies in the S2CLS fields. A cross-correlation analysis carried out on a sample of approximately 600 counterparts from the UKIDSS Ultra Deep survey led to an estimation of the halo masses of these SMGs and a comparison with passive and star-forming galaxies selected in the same field. It was found that, on average, the SMGs occupy high-mass dark matter halos (*M*_*halo*_>10^13^ M_⊙_) at redshifts *z*>2.5, consistent with being the progenitors of massive elliptical galaxies found in present-day galaxy clusters. It was also found that the SMG clustering strength was consistent with that of the star-forming population and that this appears to be the case across all redshifts. Recent work by Bourne *et al*. [131] explored the evolution of cosmic star formation in the S2CLS data sample. They concluded that the star formation history appears to undergo a transition at *z*∼3–4, as unobscured structure growth in the early Universe is surpassed by obscured star formation, driven by the gradual build-up of the most massive galaxies in the Universe during the peak of cosmic assembly. The S2CLS catalogue and images have also presented an opportunity for follow-up work, both in terms of the properties of individual sources (e.g. with ALMA) and also in the statistical analysis of the entire sample.

#### 4.1.5. SASSy: the SCUBA-2 all-sky survey

Prior to the start of the legacy survey observing campaign, the original ‘all-sky’ survey (SASSy) was reborn as the more modest ‘SCUBA-2 Ambitious Sky Survey’, with the aim of making the largest submillimetre map of the Outer Galaxy to identify the coldest and earliest regions of star formation. As described by Thompson & Gibb [132], the survey would cover of order 700 degree^2^ between longitudes 120° and 240°, extending to ±2° from the Plane. The primary goal was to detect all the compact sources within the survey bounds above a few times the approximately 40 mJy noise level at 850 μm. The survey was allocated 480 hours of mainly poor weather (band 5) observing time, and was often used as a fallback project in poor weather conditions. The sources identified are in the process of being compared to the *IRAS* and *Planck* catalogues to determine if any new objects have been detected. For example, in the 120 degree^2^ region of the Galactic Plane covering longitude 120°<*l*<140° and latitude |*b*|<2.9°, Nettke *et al.* [133] produced a catalogue of approximately 300 sources, of which 19 were new detections in comparison to *IRAS*, 41 new detections compared to *Planck* and 13 that were not found in either catalogue. Analysis continues of this very extensive dataset.

#### 4.1.6. SCUBA-2 Observations of Nearby Stars survey

Although the main strength of SCUBA-2 is in wide-field mapping, the camera can also image compact sources very quickly and with high image fidelity. The SCUBA-2 Observations of Nearby Stars survey (SONS) therefore sought to capitalize on the pioneering work of SCUBA (as described in §3.4) by targetting 100 nearby stars, looking for evidence of debris discs—the extrasolar analogues of the EKB in our Solar System. As described by Matthews *et al.* [134], the survey aimed to characterize these discs by: (i) providing direct dust masses that could not be obtained from shorter wavelengths alone; (ii) adding to the far-IR/submillimetre spectrum to constrain the dust size distribution; (iii) using the power of a 15 m telescope to resolve disc structures around the nearest systems; and (iv) looking for evidence of resonant clumps and other features in resolved structures that could be indicative of unseen perturbers, such as planets. Of particular importance was to investigate the diversity of exo-planetary system architectures, as this represents a key piece of information that will help link the formation and evolution of planetary systems with the evolution of planetary building blocks (planetesimals). The survey used 325 hours of observing time and for the 100 targets reached an average 1*σ* noise of approximately 1.2 mJy beam^−1^. A total of 49 discs were detected, many for the first time, and 16 of the nearest discs were also spatially resolved by the JCMT.

**Figure 15.**
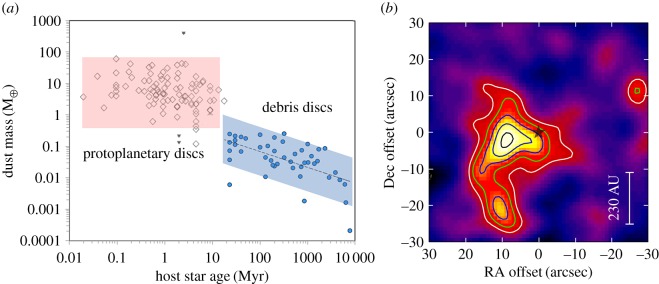
(*a*) The derived dust mass as a function of host star age, for both protoplanetary and debris discs (the latter exclusively from the SONS survey). (*b*) The SONS 850 μm image of the debris disc surrounding the nearby Sun-like star HD 38858. The dust mass figure was adapted from Matthews & Kavelaars [135] and includes the results from the SONS survey. The HD 38858 figure is adapted from Holland *et al.* [136].

The results from SONS, presented by Holland *et al*. [136], more than doubled the number of imaged discs from submillimetre observations. The discs are characterized in terms of their flux density and size (radial distribution of the dust), and derived dust properties from their spectral energy distributions. The mass of a disc, for particles up to a few millimetres in size, is uniquely obtainable from submillimetre observations, and shows a slow decline with age over hundreds of millions of years of stellar evolution (figure 15*a*). Many individual objects from SONS have also been studied with some surprising results. For example, observations of the nearby Sun-like star HD 38858 revealed a large, extended structure, clearly with a flux peak offset from the star position (figure 15*b*). Kennedy *et al.* [137] used multiple wavelength data, including from *Herschel*, to determine that, although the disc is clearly resolved by the SONS observations, the peak to the south is most probably a background object. The offset nature of the peak emission is still puzzling, but the emission might indicate a perturbed disc that could have detectable volatiles. Similarly, observations of the nearby main sequence star 61 Vir, a system which has at least two known inner planets, reveal a resolved disc with a diameter of at least 80 AU, i.e. very similar to the EKB in our Solar System. Marino *et al*. [138] combined the SCUBA-2 data with ALMA observations to conclude that the disc is very likely extended from 60 to over 100 AU and so represents a very broad parent planetesimal belt. The observations of 61 Vir have already shown the legacy of the SONS survey, in that it is providing a comprehensive target list for high angular resolution follow-up observations with submillimetre interferometers, such as SMA an ALMA.

### 4.2. The East Asian Observatory era: a new call for large programmes

On 1 March 2015, the East Asian Observatory (EAO) officially took over responsibility for the operations of the JCMT. Shortly after this (1 July), there was also a second call for ‘Large Programmes’ to be undertaken with the JCMT, covering the period from late 2015 until late 2018. A total of seven programmes were approved (six would use SCUBA-2) and observations began in November 2015. Some 50% of the total telescope time, amounting to at least 2400 hours, was to be dedicated to these programmes. A second large programme call for either extension to the existing ones or new initiatives was issued in February 2017.

#### 4.2.1. Studies: the SCUBA-2 ultra deep imaging EAO survey

The objective of STUDIES is to obtain the first confusion-limited 450 μm map, centred on the COSMOS field at the northern edge of the CANDELS region [139]. The single pointing (Daisy map) of around 10 arcmin in diameter will eventually use 330 hours of the best weather available on Mauna Kea. This will take advantage of the high angular resolution offered by the JCMT/SCUBA-2 at 450 μm, compared to *Herschel* at 350 and 500 μm, and allow detections of faint galaxies with a significantly higher surface density. The goal is to reach a 1*σ* noise of 0.6 mJy beam^−1^ at the centre of the field and to detect the dominant members in the dusty galaxy population that give rise to the bulk of the far-IR extragalactic background. Such a deep map will enable the detection of nearly all L_*IR*_>10^12^ L_⊙_ galaxies at *z*<4, and the majority of L_*IR*_>10^11^ L_⊙_ galaxies at *z*<2. The observations will also allow, for the first time, a substantial overlap in the star formation rate range with galaxies detected by deep optical surveys. This will provide a more complete census of the cosmic star formation that is both obscured and unobscured by dust. In just over a year (November 2015–February 2017) the observations were 40% complete, and have reached 1 mJy beam^−1^ in the centre of the image. A total of 98 sources have been detected so far at a significance of greater than 4*σ*, with this number expected to dramatically increase as the map goes deeper [139].

#### 4.2.2. SC2-COSMOS

**Figure 16.**
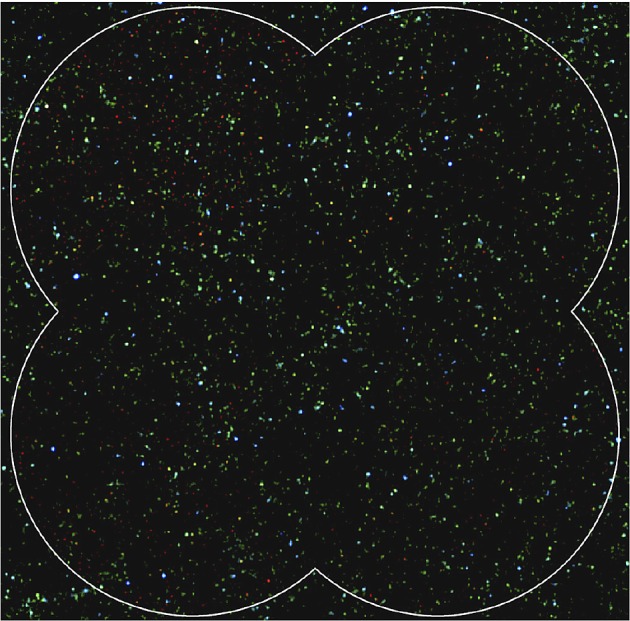
True colour image of the COSMOS field created from SCUBA-2 850 μm (red) and *Herschel*/SPIRE 250 μm (blue) and 350 μm (blue). The map highlights the rare dusty sources which are most probably some of the highest redshift galaxies in the sample. The white line represents the coverage of the 1.4×1.4° SC2-COSMOS map. Adapted from Matsuda [140].

COSMOS is a survey of approximately 1000 submillimetre galaxies in the 2 degree^2^ COSMOS field [140]. The region is the pre-eminent ALMA-visible, degree-scale, extragalactic survey field, and has been studied extensively from the X-ray to the radio. The goal is to first complete the 850 μm map of the full COSMOS field (partly covered by SCUBA-2 Cosmology Legacy Survey) to a depth of 1.5 mJy beam^−1^, and to then increase the depth of this map to 1.2 mJy. This map will have twice the area of similar surveys in a single contiguous field, allowing unique tests of the clustering of the submillimetre galaxy population on scales up to approximately 60 Mpc. By March 2017, the observations were 79% complete against an allocation of 223 hours, and a 1*σ* noise of less than 1.5 mJy beam^−1^ has been achieved across the entire field (as shown in figure 16). Already, some 1400 submillimetre sources have been detected in the field and an analysis of the multi-wavelength properties of these galaxies is underway. Follow-up observations with ALMA of the 150 brightest sources are also planned.

#### 4.2.3. JINGLE: the James Clerk Maxwell Telescope dust and gas in Nearby Galaxies Legacy Exploration

JINGLE is a survey designed to systematically study the cold ISM of galaxies in the local Universe [141]. The survey will provide 850 μm images with SCUBA-2 for a sample of 192 *Herschel*-selected galaxies, as well as integrated CO(2-1) line fluxes with the heterodyne Receiver A (RxA) for a subset of 62 of these galaxies. A total of 780 hours has been allocated to the survey over the 3-year period. The sample builds on multiple surveys including *Herschel*/H-ATLAS and the MaNGA optical integral-field spectroscopy surveys. By combining the results from the JCMT observations with all these ancillary data, JINGLE will allow for a detailed characterization of the gas and dust properties of galaxies in the local Universe. Scientific objectives include studying the dust-to-gas ratio and how it varies across the galaxy population, correlating the molecular gas content with spatially resolved galaxy properties, and investigating the correlation between ISM properties and the dynamics of galaxies. The scaling relations between dust, gas and global physical properties will also provide critical benchmarks for high-redshift studies with JCMT and ALMA. As of early 2017, the survey is 36% complete with 106 galaxies already observed with SCUBA-2.

#### 4.2.4. BISTRO: B-fields in star-forming region observations

Without accurate knowledge of the collapse process of molecular clouds, it is not possible to understand how a star forms. The exact role of magnetic fields (B-fields) in this process is still open to considerable debate, and so the BISTRO survey will address this by tracing the direction and strength of the magnetic field on scales of approximately 1000–2000 AU in the central regions of several nearby molecular clouds (e.g. Orion, Ophiuchus, Taurus L1495) already observed with the previous Gould Belt legacy survey [142]. The scientific objectives are to assess the relative importance of magnetic field and turbulence in the star formation process, to test models of magnetic ‘funnelling’ of materials onto filaments and to investigate the role of B-fields in shaping protostellar evolution (including bipolar outflows from young protostars). The survey uses a rotating half-waveplate polarimeter that was developed specifically for use with SCUBA-2 [143] and was awarded a total observing time of 224 hours of good weather (band 2). As of early 2017, some 38% of the programme has been completed, with regions such as Orion A, Ophiuchus and Serpens Main already observed. An example of one of the early observations is shown in figure 17 from Ward-Thompson *et al*. [144] for the central region of the Orion molecular cloud. The image shows that magnetic field lies perpendicular to the famous ‘integral-shaped filament’ and may be responsible for ‘funnelling’ matter onto filaments to aid the formation of dense cores that eventually become protostars.

**Figure 17.**
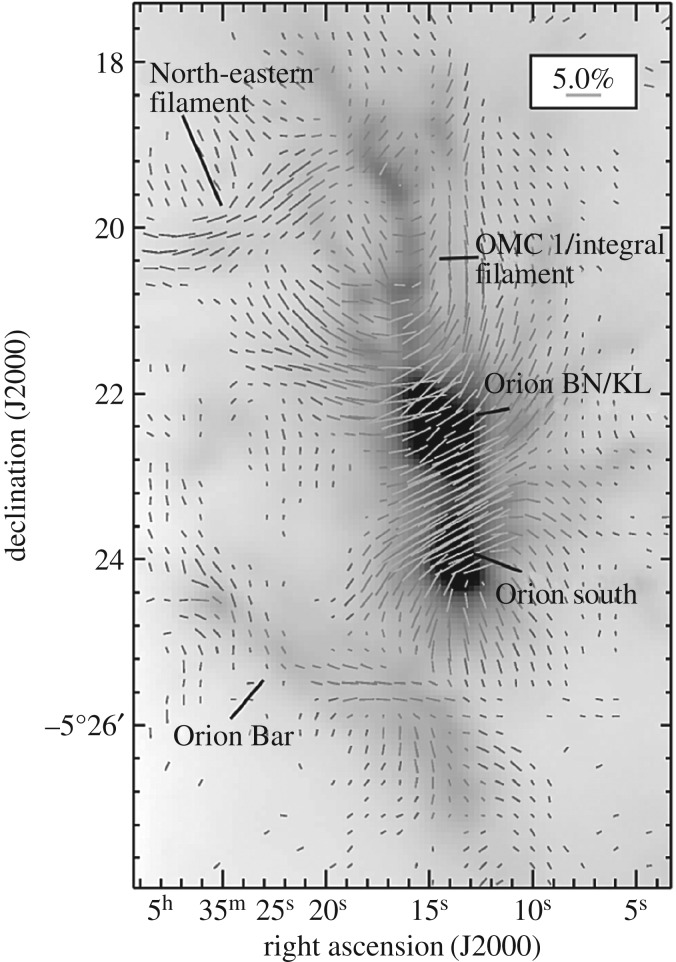
850 μm Polarization map of the central region of the Orion molecular cloud, in which the measured polarization vectors are rotated by 90° to show the B-field direction. Adapted from Ward-Thompson *et al.* [144].

#### 4.2.5. SCOPE: SCUBA-2 continuum observations of pre-protostellar evolution

It is known that stars form in the densest regions within molecular clouds, the earliest phase being linked with the so-called pre-stellar cores. However, the formation and early evolution of these cores in different environments is not well known. The SCOPE survey is carrying out an ‘all-sky’ survey at 850 μm of a sample of 2000 cold clumps identified by the *Planck* surveyor [145]. The JCMT/SCUBA-2 is more sensitive to cold dust than *Herschel*, and also has high angular resolution to resolve the substructure of *Planck* cold clumps. The aims of the survey include the study of how dense cores form and how star formation varies as a function of environment, the universality of filaments in the cold ISM and their roles in generating dense cores, how dust properties change in different environments and how dust properties affect the chemical evolution of dense cores. The survey is being supplemented with observations from other millimetre and radio telescopes (such as Purple Mountain Observatory and the Nobeyama Radio Observatory 45 m telescope), and will also form a legacy database for such studies with other instruments, especially ALMA. The survey was awarded 300 hours of band 3 and 4 time, and as of March 2017 was 69% complete.

#### 4.2.6. TRANSIENT: a transient search for variable protostars: how do stars gain their mass?

The TRANSIENT programme is using SCUBA-2 to measure accretion variability in protostars in eight fields within nearby star-forming regions [146]. It has been found that an outburst in accretion luminosity heats the dust in the envelope, which is then seen as brighter emission at 850 μm [147]. Far-IR and submillimetre observations provide a snapshot of accretion rate, averaged over the few-weeks heating timescale for the luminosity burst to propagate through the envelope. Given the difficulty of the carrying out such long-term observations in the far-IR (the need for a space-based observatory), submillimetre monitoring may be the only way to probe the earliest stages of stellar growth, because these stars are so heavily embedded that they are not visible at optical/near-IR wavelengths. The monitoring programme includes a total of 182 embedded protostars (Class 0/I YSOs with envelopes) and 800 disc/flat-spectrum objects. Each region will be observed once a month for a total period of 3 years to search for signs of variability across epochs. The programme was awarded 150 hours of observing time, split equally in weather bands 1, 2 and 3. As of March 2017 the programme is 27% complete, and the first variable candidate has already been identified.

### 4.3. Other science highlights from SCUBA-2

#### 4.3.1. Observations of Comet ISON

In late 2013, the JCMT launched a campaign to study the chemistry of the sun-grazing comet C/2012 S1 (ISON). Several groups of observers concentrated on measuring the production rate of HCN and water as the comet approached perihelion. The comet was also observed multiple times with SCUBA-2, catching the final hours before it disintegrated. Keane led the team on these observations [148], which showed that as ISON approached perihelion, the continuum emission from the nucleus became an elongated dust column spread out over 60 arcsecs (approx. 10^5^ *km*) in a direction away from the Sun. One of the final images reveals distinct clumps, consistent with the catastrophic disruption of the comet, producing approximately 5×10^10^ kg of millimetre-sized dust.

#### 4.3.2. New insights on planet formation

SCUBA-2 has also been used to explore and place new constraints on the dust and gas mass of protoplanetary discs during the giant planet building phase. Williams *et al*. [149] surveyed a half-degree field towards the *σ* Orionis cluster, which contains almost 300 YSOs with estimated ages of 3 Myr. Only nine stars were detected from the observations at 850 μm, with these having estimated disc masses of between 5 and 17 Jupiter masses. Using a stacking analysis, the mean mass for 83 infrared-detected objects that were not detected by SCUBA-2 was determined to be 0.5 Jupiter masses, effectively ruling them out of ongoing planet formation. The lack of emission illustrates how little raw material must remain in the environs of the vast majority of these young objects. This suggests that planet forming must start very early on, and that the growth of planetary cores must be largely complete within a couple of Myr after the host star becomes optically visible.

#### 4.3.3. Dust surrounding a pulsar

The nearby Geminga pulsar is believed to have crossed the Galactic Plane in the last 100 000 years. Greaves & Holland [150] report the detection of a shell of material surrounding Geminga that could have formed from compression of the local interstellar medium. A compact source is detected from 450 μm observations which may be evidence for the existence of a circum-pulsar disc, the first time any such structure has been detected in the submillimetre region. The inferred mass of dust is expected to exceed six Earth masses, and so has the potential to form low-mass planets such as the archetypes around PSR B1257+12 [151]. Further imaging at high angular resolution is planned for this object.

#### 4.3.4. Embedded binaries and their dense cores

The relationship between young, embedded binary stars and their parent cores is not well understood. Sadavoy & Stahler [152] used VLA and SCUBA-2 observations of a number of young stars and cores in the Perseus molecular cloud to explore the origin of binary stars. It was revealed that most embedded binaries are found towards the centres of their parent cores. Wide (greater than 500 AU separation) binaries tend to be aligned with the long axes of the core, while tight systems show no preferred orientation. The authors tested a number of evolutionary models in an attempt to account for the populations of both single and binary Class 0 and I sources. The model that best fits the observations suggests that all stars form initially as wide binaries, and then either break up into separate stars or shrink into tighter orbits. Future observations will explore whether the high mass fraction of dense cores that become stars in Perseus is similar in other star-forming regions.

#### 4.3.5. SUPER GOODS: ultra-deep imaging of the GOOD-N field

In addition to the extensive surveys that formed the Cosmology Legacy Survey, Cowie *et al*. [153] carried out ultra-deep imaging with SCUBA-2 of the GOODS-N field. The maps, covering 450 arcmin^2^, detected 31 and 186 sources at 450 and 850 μm, respectively, and reached sensitivity levels well below the confusion limit at 850 μm. Using extensive VLA and SMA observations to pinpoint exact galaxy locations, and Keck spectra to determine redshifts, it was shown that the star formation rate of these galaxies reaches a peak at *z*=2–3, before dropping at higher redshifts. It was also suggested that the shape in the number density of galaxies per unit volume as a function of star formation rate is invariant over this particular redshift range.

#### 4.3.6. The space density of galaxies at *z*>4

Until the advent of *Herschel*, only a handful of dusty star-forming galaxies were known to exist at redshifts greater than 4 and most of these were amplified by gravitational lensing. Ivison *et al*. [154] selected 109 galaxies for SCUBA-2 imaging based on their extremely red far-IR colours and faint 350 and 500 μm fluxes from the *Herschel*-ATLAS imaging survey. The addition of the submillimetre data allowed the peak of the spectral energy distribution to be identified and so led to better constraints on the redshifts of these objects. The galaxies were determined to be in the redshift range 3.3–4.3 (median value of 3.66), with a third lying at *z*>4, suggesting a space density of approximately 6×10^−7^ Mpc^−3^. The sample contains some of the most luminous star-forming galaxies and the most overdense cluster of early starburst ellipticals known to date.

## 5. Science with the James Clerk Maxwell Telescope heterodyne instrumentation

At the time of the dedication of the JCMT in March 1987, two heterodyne receivers were in operation on the telescope; namely a polarization-splitting dual-channel 230 GHz band receiver (RxA) [155] and a single-channel 345 GHz band receiver (RxB) [5,6]. Both receivers were equipped with Schottky diode mixers. The 230 GHz receiver, RxA, covered the range 220–280 GHz using two sets of mixers—one set for the lower part and one for the upper part of the band. RxA was operated for a short time as a dual-channel receiver, but for most of the time operated in a hybrid mode with mixers in the opposite polarization covering different frequency ranges. The 345 GHz receiver covered the range 320–370 GHz and used a carcinotron as the local oscillator (LO) source. An early goal was to equip the telescope with state-of-the-art single- or dual-pixel SIS receivers and subsequently array receivers with a priority for the 345 GHz band. This was made more feasible with Canada joining the UK/Netherlands project in the spring of 1987, injecting additional resources into the JCMT instrument development fund. A number of single-feed SIS receivers were deployed in the early part of the 1990s followed by polarization-splitting, dual-channel receivers.

**Figure 18.**
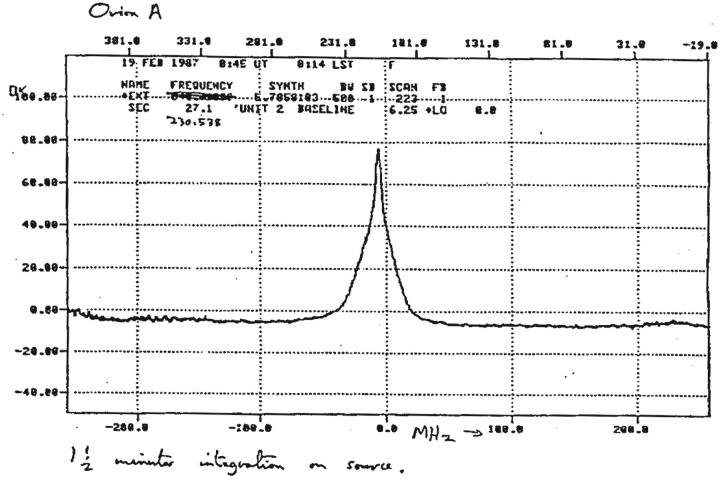
One of the first spectra obtained by the JCMT. A CO *J*=2–1 spectrum of OMC1 with RxA from January 1987. Figure from the third issue of the JCMT newsletter PROTSTAR (ISSN 0267-1247).

In the early years, heterodyne science observations were constrained to single-point spectra (as shown in figure 18) or making small maps up to a few arcminutes in size. Observing larger areas was too time-consuming for the single-pixel instruments with their limited sensitivity and spectrometer dump time. The first change occurred in 1992 with the introduction of the DAS spectrometer [7] allowing dump times to be reduced to a second and the associated development of ‘on-the-fly’ software for heterodyne mapping. This, combined with more sensitive instruments, caused a noticeable increase in the data rate and generated a need for more storage space. In 2007, with the Heterodyne Array Receiver Programme (HARP) [8] the JCMT became the premier observatory for mapping lines in the 350 GHz band. HARP has an array of 16 mixers, each spaced by 30 arcsecs on the sky, and was supported by a 16-channel auto-correlation spectrometer (ACSIS) [8] capable of observing a 2 GHz bandwidth in each pixel. HARP is now used extensively for large programmes and is still, after 10 years of operation, a very competitive instrument.

In parallel with the development of receivers by the partners, agreements were also made with external groups to bring receivers to the telescope. A 345 GHz SIS receiver from Ed Sutton’s group (UC Berkeley) was brought in for two short periods in 1989 and 1990. A 600 and 800 GHz receiver from Reinhard Genzel’s group (MPIA Bonn) was used in each year from 1988 to 1996 [10,11], while the SPIFI [13] visited the JCMT for short periods during 1999–2000 from Gordon Stacey’s group at Cornell. Although a few upgrades were carried out, no new heterodyne projects were started after 2000, and a high-frequency receiver project (RxE) was cancelled due to budget constraints. The receiver and spectrometer deployments are summarized in tables 1 and 2, respectively.

**Table 1.** Spectroscopic receiver instrumentation for the JCMT.

	frequency range		mixer/detector	
name	(GHz)	feeds/pixels	technology	operation period
RxA1	211–280	1^*a*^	Schottky	1987–1992
RxA2	211–280	1	SIS	1992– 1998
RxA3	211–276	1	SIS	1998–
RxB1	320–380	1	Schottky	1988– 1990
Sutton	320–380	1	SIS	1989– 1990
RxB2	320–380	2	Schottky	1990– 1991
RxB3i	320–380	1	SIS	1991– 1997
RxB3CU	320–380	2	SIS	1997– 2006
HARP	325–375	16	SIS	2007–
RxC1	460–495	1	HEB	1989–1993
RxC2	450–495	1	SIS	1993–1998
RxW	430–500^*b*^	2	SIS	1998–2014
	630–705	2	SIS	1998–2014
RxG	600/800^*c*^	1	Schottky	1988–1994
RxG2	460–495	1	SIS	1994–1996
SPIFI	630–700	25	Silicon bolometers	1999–2000

^*a*^RxA1 was a dual-channel, polarization-splitting receiver normally operated with a low-frequency mixer (220–240 GHz) in one channel and a high-frequency mixer (240–280 GHz) in the other. This effectively made the receiver a single feed at any given frequency.^*b*^The 430–500 GHz mixers were replaced with 325–375 GHz HARP mixers in 2007 to support operation with the SMA at 345 GHz.^*c*^RxG operated in the 600 and 800 GHz bands with a far-IR laser LO system. Using different gases as the lasing medium allowed tuning to a number of scientifically important frequencies.

**Table 2.** Backend spectrometers for the JCMT.

name	type	IF inputs	operation period
Kent	correlator	1	1987–1988
AOSD	AOS	1	1988–1989
AOSC	AOS	1	1989–1993
DAS	correlator	8	1992–2006
ACSIS	correletor	16	2006–

### 5.1. Chemistry

Chemistry is an integral part of heterodyne line observing: without an understanding of the chemistry, the data cannot be exploited to the fullest extent. An example is the much-studied conversion from observed CO intensity to the total molecular mass. Thus, many papers study chemistry in different regions such as galaxies, hot cores, protostellar envelopes and discs, shocks and evolved stellar envelopes. This not only gives a better understanding of the chemistry but also an improved knowledge of the source morphology and physics. The information from spectral scans aims to catalogue molecules, lines and abundances, typically in well-known objects, and often without extensive modelling or analysis of the physical conditions. There have been 10 spectral line surveys published using JCMT data covering a large fraction of the 230, 345, 460 and 650 GHz windows. Most of the surveys were conducted in the 1990s using single-pixel receivers, and groups, for example, led by Greaves [156], Sutton [157,158] and MacDonald [159] studied sources including OMC1, Sgr B2, W3, G34.4, IRAS 16293–2422 and IRC+10216. The citation count is still steadily increasing for these papers, demonstrating their great legacy value. The JCMT Spectral Line Legacy Survey (SLS) by Plume *et al*. [160] was designed to study and catalogue the lines in some typical regions—a low-mass core (NGC 1333 IRAS 4), three high-mass cores spanning a range of star-forming environments and evolutionary states (W49, AFGL 2591 and IRAS 20126), and the Orion Bar photo-dissociation region. The SLS used HARP and in contrast with most spectral surveys, a region around the central source was also observed, thereby giving additional information about the morphology and chemical variations in the local environs. In their SLS paper on the Orion Bar, van der Wiel *et al.* [161] find that the molecular abundances, in general, followed the layered structure as predicted by models of photo-dissociation regions, but there were also discrepancies between the models and the observations.

Chemistry and physics in particular regions and/or molecular species have been the topic of a large number of papers. Two such papers by van Dishoeck *et al.* [162] and van der Tak *et al.* [163] have well over 200 citations each. The paper by van Dishoeck includes a spectral scan of IRAS 16293–2422 and uses the observations to isolate the physical regions around the source with different physical and chemical properties. Results for sulphur and silicon species in the same source were reported by Blake *et al*. [164], and a similar study of IRAS 16293–2422 using more extensive JCMT and IRAM observations was published by Caux *et al*. [165]. The paper by van der Tak studied the structure around high-mass YSOs using a number of spectral lines as well as continuum and mid-IR data. The data were used to delineate the physics and chemistry in the different parts of the envelope, where freeze-out of CO was demonstrated as well as grain evaporation in the inner region. The chemistry and photo-ionization of the Orion Bar were studied by Hogerheijde *et al.* [166] and van der Werf *et al.* [167], and these papers resolved not only some of the stratification observed in the earlier-mentioned SLS paper, but also concluded that the gas in the bar must be clumpy.

Sulphur chemistry in hot cores was studied by Hatchell *et al.* [168] who found that abundance ratios of the major sulphur species did not vary between different hot cores and, with the exception of carbonyl sulphide (OCS), were in agreement with models. Hence, no evolutionary sequence was found for hot cores. Van der Tak & van Dishoeck [169] used the H^13^CO^+^ abundance to constrain the cosmic ray ionization rate in the envelope of YSOs: cosmic ray ionization forms H_3_^+^, which is then destroyed by reaction with CO in molecular regions forming HCO^+^. Models of gas–grain chemistry were tested by van der Tak *et al*. [170] by observing H_2_CO and CH_3_OH towards massive YSOs; a large number of lines allowed excitation temperatures and abundances to be determined. The CH_3_OH/H_2_ abundance shows a jump from 10^−9^ to 10^−7^ that could be attributed to grain evaporation due to radiation based on a corresponding jump in excitation temperature and correlated (IR-measured excitation) temperature of C_2_H_2_. Schöier *et al.* [171] observed IRAS 16293–2422 and derived a detailed temperature and density structure, and a detailed comparison of the observations with models strengthened the evidence for infall in the envelope. Furthermore, the molecular species are divided into those that have constant abundance in the envelope and those that have increased abundance close to the core. The later molecules, like CH_3_OH, SO and SO_2_, increase in abundance where the temperature can thermally evaporate molecules from grains. Low-mass YSOs, such as IRAS 16293–2422, have hot cores but the chemical timescales are much shorter than in the hot cores of massive YSOs.

Organic molecules were observed towards T Tauri and Herbig Ae stars by Thi *et al.* [172]. The detections showed that the emission was from dense gas at moderate temperature with some species, such as CN, enhanced by photo-dissociation. This is consistent with accretion disc models with a cold mid-plane having chemistry affected by freeze-out onto grains, while molecules formed by photo-dissociation are enhanced on the disc surface by radiation from the central object. The *ortho/para* ratio in H_2_ was been studied by Pagani *et al*. [173] using N_2_D^+^, N_2_H^+^ and *ortho* H_2_D^+^ lines. The *ortho/para* ratio in H_2_ is important for understanding the deuteration amplification in the clouds. Under some conditions the abundance of HDCO and CH_2_DOH has been found to be higher than their un-deuterated analogue species, showing a deuterium enhancement of 10^6^ times the D/H ratio. This only occurs if molecules like CO are heavily frozen out onto grains. Hence, deuterium enrichment is important for understanding and studying the freezing out of molecules onto grains. Frozen-out deuterium-enriched molecules serve as a marker of pristine material in comets.

### 5.2. Extragalactic sources

About 150 papers studying galaxies using JCMT heterodyne data have been published, and not unexpectedly, many of the these papers use the 346 GHz CO *J* = 3–2 line, which is easily observable during typical Mauna Kea weather conditions. An early example is the paper by Devereux *et al*. [174] that reported on observations of CO(3–2) in the centre regions of starburst galaxies. They found that the ratio between the CO(3–2) and CO(1–0) lines was higher in the centre of starburst galaxies than in Galactic molecular clouds, while the gas mass was typically 10% of the total dynamical mass. To explain the difference to Galactic molecular clouds from earlier studies, the larger ratio required a more complicated model than just one in which the gas was hotter. Yao *et al.* [175] extended these investigations and observed 60 IR-luminous sources selected from the SCUBA Local Universe Galaxy Survey (SLUG). The authors reported an almost identical average CO(3–2) to CO(1–0) line ratio but with a much larger spread in values, indicating a large variation in excitation of the gas in IR-luminous galaxies. In parallel, higher-level CO lines as well as lines from HCN, HCO^+^, HNC^+^ and CS were used to study the gas excitation in starburst and IR-bright galaxies. The CO(6–5) transition, observed by Harris *et al.* in 1991 [176] using Receiver G, clearly showed that the gas in nearby starburst galaxies such as M 82 and NGC 253 was hotter and denser than in typical Galactic clouds. Such studies were extended to high-redshift starbursts by Papadopoulos *et al*. [177] with the detection of CO(4–3) in galaxies at redshifts of 3.79 and 3.53. A number of possible sources for the excitation were discussed in these papers, such as violent turbulence, the presence of OB stars or cosmic rays due to AGNs. Bradford *et al.* [178] concluded that, in the case of NGC 253, the CO(7–6) emission observed using SPIFI was excited by cosmic rays due to the high supernova activity in the region.

Aalto *et al.* [179] discovered an unexpectedly high HNC/HCN line ratio in star-forming galaxies. In Galactic warm dense gas, this ratio is lower, even in photo-dissociation regions. The observed high line ratio was explained by IR excitation of HNC, which has a much lower energy-bending mode than HCN, or, alternatively, by X-ray-dominated chemistry due to the presence of AGNs. The paper by Greve *et al.* [180] studied the starburst galaxies Arp220 and NGC 6240 in several molecular species and transitions. The authors showed that the emission from these molecules traced different densities and there is a size-density relationship for the gas, similar to, but steeper than that observed in Galactic clouds. The bulk of the gas mass approximately (1–2)×10^10^ M_⊙_ resides in a dense *n*=10^5^–10^6^ cm^−3^ warm phase. Papadopoulos *et al.* [181] presented spectral line energy distributions for 70 LIRGs, with the galaxies covering a range of infrared luminosities and morphologies showing a broad range of ISM conditions. On the high excitation side, the ISM is dominated by hot (greater than 100 K) and dense (*N*>10^4^ cm^−3^) molecular gas with gas mass reservoirs of approximately (few) 10^9^ M_⊙_. The authors conclude that the gas excitation in merger-driven ULIRGs is dominated by turbulence and cosmic rays rather than UV/optical photons and supernova shocks. This new understanding of the gas phase in massive star-forming galaxies was used to guide later observations with the *Herschel* satellite and ALMA. Another noteworthy and highly cited galaxy paper was presented by Edge [182], who showed that hot gas in galaxy clusters, cooled by X-ray emission, generates a cooling flow of gas onto the galaxies in the clusters. Searches for CO in the central galaxies of clusters with cooling flows had only provided one detection before Edge reported 16 more detections of CO in galaxies at the centre of clusters with cooling flows.

The NGLS (see §iii) observed an HI-selected sample of 155 galaxies spanning all morphological types with distances less than 25 Mpc. The survey has so far produced 10 papers, e.g. Wilson *et al.* [183]. The objective of the heterodyne component of the survey was to study the gas properties and gas-to-dust ratio, and to compare radial profiles of the dust, HI and CO emission. The authors find a wide range of molecular gas mass fractions in the galaxies in the sample. By comparing the NGLS data with merging galaxies at low and high redshift, which have also been observed in the CO *J*=3–2 line, they show that the correlation of far-IR and CO luminosity shows a significant trend with luminosity. This trend is consistent with a molecular gas depletion time that is more than an order of magnitude faster in the merger galaxies than in nearby normal galaxies. There is also a strong correlation of the *L*_*far*−*IR*_/*L*_*CO*(3−2)_ ratio with the atomic-to-molecular gas mass ratio. This correlation suggests that some of the far-IR emission originates from dust associated with atomic gas and that its contribution is particularly important in galaxies where most of the gas is in the atomic phase.

### 5.3. Clouds, cores and galactic structure

Observing and mapping molecular clouds and cores in CO or other lines is a common topic for JCMT heterodyne papers, with around 150 papers having ‘cloud’ and/or ‘core’ in their title. Some well-cited examples include the paper by Davis *et al.* [184] who mapped the Serpens molecular cloud in the CO (2–1) line and continuum, identifying cores and outflows and estimating their ages. The paper by Kirk *et al*. [185] studied the kinematics of dense cores in the Perseus molecular cloud with the N_2_H^+^ (1–0) and CO (2–1) lines, and found that the internal motion measured by the N_2_H^+^ line-width in the SCUBA-selected dense cores was more than sufficient to support against gravitational collapse. While many cloud regions were mapped early on with the JCMT heterodyne instruments, large-scale mapping did not start until 2007. The change was triggered by the decision to allocate large amounts of time to large surveys and the arrival of HARP/ACSIS made this feasible on the heterodyne side. This shift in policy was instigated to keep the JCMT competitive in the era of large submillimetre interferometers as the superior angular resolution from the latter would make them much better suited to observe compact objects, such as protostellar accretion discs.

**Figure 19.**
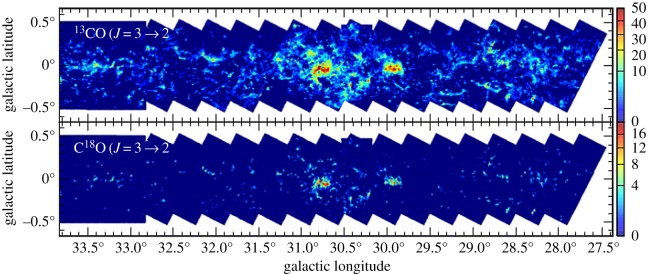
Part of the CHIMPS survey of the Galactic Plane. (*a*) Velocity integrated emission of ^13^CO(3–2) and (*b*) the same for C^18^O(3–2). Adapted from Rigby *et al.* [196].

The first-generation JCMT Legacy surveys began in 2007, concentrating on heterodyne observations (SCUBA-2 would join the surveys, but not until 2012, as discussed in §3). Of these surveys, SLS and NGLS have already been described, with a third major survey concentrating on mapping the extent of ^13^CO and C^18^O(3–2) in a number of molecular clouds in the Gould Belt. The results from these surveys have been published in a series of papers, including the Orion B region by Buckle *et al.* [186], the Perseus molecular cloud by Curtis *et al*. [187], Taurus by Davis *et al.* [188], Serpens by Graves *et al*. [189], Orion A by Buckle *et al.* [190] and the Ophiuchus region by White *et al*. [191]. As an example of the results, the Buckle *et al.* paper [186] presents temperature, opacity, mass and energy content and location of outflow regions in Orion B. Several follow-up papers, including those from Curtis *et al.* [192] and Drabek-Maunder *et al*. [193], went on to analyse the data further in terms of detailing the extent and properties of outflows in the regions. In addition, the Perseus region was mapped in the dense gas tracers HCO^+^ and HCN by Walker-Smith *et al.* [194]. Outside of the formal legacy surveys, additional surveys included the CO High Resolution Survey (COHRS) by Dempsey *et al.* [195], which mapped the Galactic Plane in CO(3–2) within the area 10<*l*<65 and |*b*|<0.5. The ^13^CO/C^18^O(3–2) Heterodyne Inner Milky Way Plane Survey (CHIMPS) published by Rigby *et al.* [196] covers the Galactic Plane area 28<*l*<46 for |*b*|<0.5 (figure 19). All of the surveys were obtained with a spatial resolution of 14 arcsecs and are publicly available: part of the JCMT’s legacy for the future.

### 5.4. Star formation: outflows and discs

Spectroscopy of specific star formation regions (as opposed to entire molecular clouds described above) has also been an extensive area of research for the JCMT. Around 200 papers based on JCMT heterodyne data have ‘star formation’, ‘outflow’ or ‘disc’ in the title. The papers cover many aspects from compact accretion discs and cores to envelopes and large-scale outflows. Some the most cited papers in the area of star formation with the JCMT are discussed below.

Individual outflows have been observed to determine their morphology and physical characteristics. For example, Richer *et al.* [197] mapped the outflow in Orion B and modelled the outflow as driven by a neutral highly collimated jet, the collimation increasing with velocity in the outflow. Lada & Fich 1996 [198] observed the outflow in NGC 2264G and again found collimation increasing with outflow velocity. The outflow obeyed a ‘Hubble Law’ with the gas velocity increasing further away from the central source. The NGC 1333/IRAS4 outflow source was studied by Blake *et al.* [199], with the authors deducing a depletion of CO and other molecules in the flow, as well as observing the additional presence of SiO in the outflow. Indeed, SiO was later identified as an outflow indicator by Nisini *et al.* [200]. Large parts of the NGC 1333 cloud complex were surveyed by Knee & Sandell [201], identifying 10 protostellar sources, each of which was found to be driving an outflow. A number of survey papers addressed the issue of whether all protostellar and YSOs have outflows. The paper by Parker *et al.* [5] surveyed IRAS sources representative of low-mass YSOs embedded in dark molecular clouds and found outflows in 70% of the targets. Other surveys also detected outflows in large fractions of the targets, suggesting that many, if not all, such objects have outflows. The survey of the Perseus Cloud complex by Hatchell *et al.* [202] found outflows in 65% of the 51 SCUBA-identified cores, and all but four of the outflows were also identified by *Spitzer* as YSOs. Indeed, only one of the *Spitzer* sources did not have a detected outflow, again showing the almost complete correlation between YSO and outflows, and that a large fraction of the cores also have embedded YSOs. Outflows deposit kinetic energy into the circumstellar envelope and cloud, which has the potential to stop accretion, disrupt the envelope and generate turbulence that supports the cloud (or can even disrupt the cloud). Hence, although outflows have a clear impact on the star formation process, there is still no clear consensus of just how significant a factor this is.

Protostellar accretion discs were directly studied by using short-baseline interferometry involving the nearby Caltech Submillimeter Observatory (CSO) and the Smithsonian Millimeter Array (SMA), as discussed in §5.6. Without the sub-arcsecond resolution afforded by an interferometer, accretion discs are unresolved by the JCMT, and the disc emission needs to be disentangled from that of the ambient cloud. This can be achieved in a number of ways; for example, using the chemical or physical characteristics of the region, e.g. line width, or selecting sources that have separated from the parent cloud either spatially or in velocity space (it is not uncommon that T Tauri stars have left their parent cloud or the cloud has been disrupted). The paper by Thi *et al.* [203] studied discs around T Tauri and Herbig Ae stars using *ISO* H_2_ and JCMT CO(3–2) and CO(6–5) data, selecting sources that are spatially separated from their ambient clouds. The H_2_ emission arises from hot (100–200 K) gas, while the CO emission is from cooler (20–80 K) gas. The lower-level CO emission profile was shown to be double-peaked, characteristic of a disc in Keplerian rotation. By comparing mass estimates from the CO line and continuum, the CO abundance was found to be lower than predicted, which was attributed to freeze-out in the disc centre and photo-dissociation on the disc surface. Van Zadelhoff *et al*. [204] observed the sources LkCa 15 and TW Hya in a number of high-excitation lines, showing that the line emission mainly originated from an intermediate disc layer with high densities of 10^6^–10^8^ *cm*^−3^ and moderately warm temperatures. The authors found evidence for significant freeze-out of CO and HCO^+^ at low temperatures, but the abundance in the warmer upper layer was also low and attributed to photo-dissociation. The first detection of DCO^+^ in the disc of TW Hya was reported by van Dishoeck *et al.* [205]. The DCO^+^/HCO^+^ ratio was found to be 0.035±0.015, similar to values in pre-stellar cores. Organic molecules in protoplanetary discs surrounding T Tauri and Herbig Ae stars were studied by Thi *et al*. [172], using the JCMT for high-excitation lines and the IRAM 30 m telescope for low-excitation lines. The main conclusions were that abundances were lower compared to the envelopes around protostars. The importance of photo-dissociation is shown by the CN/HCN ratio that is found to be higher than in Galactic photo-dominated regions, which have enhanced CN/HCN abundance ratios due to photo-dissociation.

### 5.5. Solar System: planetary atmospheres and comet chemistry

The study of Solar System objects using the JCMT heterodyne instruments started with observations of the Sun during the total Solar eclipse over Hawaii on 11 July 1991. As well as continuum observations of sunspots, spectroscopy of the chromosphere during limb occultation was carried out by Lindsey *et al.* [206], resulting in a measurement of the chromospheric temperature profile in the near-millimetre region. The limited bandwidth of the heterodyne instrument reduced the problem with saturation—sensitivity was not an issue. Other Solar System observations included the first detection of CO and HCN in Neptune by Marten *et al.* [207], and of CO in the atmosphere of Pluto at millimetre wavelengths by Bockelée-Morvan *et al.* [208] and Greaves *et al.* [209]. The detection of the important catalyst H_2_O_2_ in the Martian atmosphere by Clancy *et al*. [210] was the first such detection in a planetary atmosphere outside that of the Earth. Venus is the planetary atmosphere most studied with the JCMT and a dozen papers, including those by Clancy *et al.* [211] and Sandor *et al*. [212], have reported observations of temperature structure, wind patterns through the Doppler shifts and atmospheric chemistry and its variability.

Comets are by far the most studied Solar System objects with the JCMT heterodyne instruments, having been observed and monitored from the early 1990s. An early influential paper by Senay *et al.* [213] highlighted that enough CO gas was observed to explain coma outburst in the most distant comets. For comets close to the Sun, the sublimation of water ice is a dominant driver, but the temperature in distant comets is too cold to allow sublimation to generate the outbursts. The JCMT has also monitored the gas abundance in a number of comets often in conjunction with other telescopes. The comets Hale–Bopp and Hyakutake were studied in a number of highly cited papers by Biver *et al*. [214–216]. The detection of HNC from Comet Hyakutake by Irvine *et al.* [217] was first seen as evidence for the existence of interstellar ices in comets. The HNC/HCN ratio was found to be similar to the interstellar gas phase value and higher than the equilibrium ratio expected in the outermost Solar nebula, where comets are thought to be formed. The HNC ratio was later explained by the same authors [218] as being due to photo-chemistry in the comet coma and not an indication of interstellar origin, whereas isotopic studies supported the view that comets contained pristine unprocessed interstellar ices. Meier *et al.* [219] reported the third detection of HDO in a long-period, Oort Cloud comet; the data all giving HDO/H_2_O abundances ratios about twice the terrestrial ocean values. Such detections did not support the view that comets supplied the majority of the water for Earth’s oceans. The detection of DCN in Comet Hale–Bopp by Meier *et al.* [220] revealed an even higher deuterium enrichment in HCN of approximately 6–8 times the ratio in H_2_O. Different deuterium enrichment is a hallmark of interstellar ion-neutral and grain-phase chemistry, while it is not expected in material processed in denser and warmer part of the Solar nebula. These observations were strong evidence for the presence of interstellar ices in comets.

### 5.6. Interferometry: accretion discs and supermassive black holes

There was early interest in using the JCMT as part of an interferometer, with the first experiment taking place in January 1992. This was a millimetre-wave VLBI including the JCMT, Nobeyama, SEST and OVRO. No fringes, however, were found. About the same time the first tests were carried with the Short Baseline Interferometer (SBI) between the JCMT and the CSO [221,222]. It operated at the 230, 345 and 460 GHz bands and was one of the first, if not the first interferometer operating at submillimetre wavelengths. The two-element interferometer became a forerunner of the high-frequency interferometers operating today, such as the SMA and ALMA. The main scientific contribution of SBI was the detection of accretion discs around a number of YSOs. With a resolution of 1 arcsec or better it could resolve the accretion disc down to a size of approximately 70 *AU* for nearby objects. Lay *et al.* [222] observed HL Tau and L1551 IRS5, partly resolving the sources with disc major axes of 60 AU and 80 AU, respectively, while the minor axis was constrained to be less than 50 AU. The elongation of the disc was perpendicular to the outflow, as expected. In total, 16 protostellar sources were observed including class 0, I and II YSOs, with work by Brown *et al.* [223] confirming accretion discs with masses of at least 10^−2^ M_⊙_. Wiedner *et al*. [224] also carried out the first submillimetre interferometric observations of Arp 220 in both line and continuum. The continuum emission at 342 GHz clearly was binary in the east–west direction with a separation of 1 arcsec. The CO(3–2) line showed a binary source at some velocities but a more extended structure at other velocities.

The construction of the SMA on Mauna Kea made it possible for both the JCMT and CSO to join the array of eight antennas, with the project becoming known as the extended SMA (eSMA). Such an extension would contribute significantly longer baselines while affording more sensitivity by doubling the collecting area, thus allowing the observation of weaker sources with even higher angular resolution than was possible with the SMA alone. The first science campaign occurred in April 2008, with Bottinelli *et al.* [225] reporting the first detection of CI absorption towards the lensed system PKS1830–211 at *z*=0.866. The results also showed that it was possible to resolve regions with different CI/CO ratios in the image. Other projects included observations of the inner envelope of IRC+10216 by Shinnaga *et al*. [226] where the HCN maser emission was resolved and vibrationally excited KCl masers were detected for the first time. Two regions were observed in the inner envelope; the acceleration zone *R*<5 R_*_ and a shell zone with a velocity close to the terminal expansion velocity. The shell zone extends to 30 R_*_ and has a clumpy structure in HCN(3–2) emission in the *v*=(0,1^1*e*^,0) state. Other papers reported on the formation of circumstellar discs around protostellar objects. One example was the dynamical velocity field of IRAS 16293-2422, investigated by Favre *et al*. [227] to within 50 AU from the central object, with the rotation deviating from Keplerian due to the disc mass being dynamically significant. Technical development at the SMA together with the pending arrival of ALMA led to a gradual decline in the use of eSMA.

**Figure 20.**
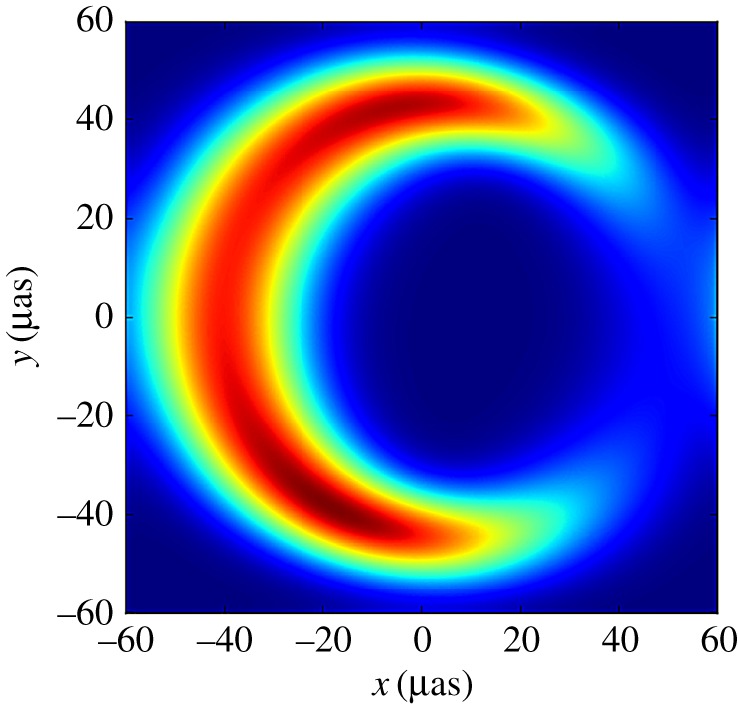
The results from a simple crescent model for the shadow of a black hole, one of a number of models being used to provide a statistical description of the existing EHT observations of Sgr A*. Adapted from Kamruddin & Dexter [228].

In April 2007, three telescopes, namely the JCMT, one antenna of the CARMA array in California and the SMT telescope in Arizona, observed Sgr A* at 230 GHz in the Galactic Centre. Doeleman *et al*. [229] reported, for the first time, on resolved structures of the size of the event horizon around the super-massive black hole (SMBH) at the centre of the Milky Way. The detected source size of approximately 40 micro-arcsecs is slightly smaller than the expected size of the event horizon of the (presumed) black hole, suggesting that the bulk of the Sgr A* emission may not be centred on the black hole, but instead arises in the surrounding accretion flow. The project to image the shadow of the black hole at the Galactic Centre was later named the Event Horizon Telescope (EHT). Up until 2013, only the three original telescopes participated in the EHT, but subsequently a phased SMA joined in, and currently eight telescopes are involved in these very long baseline interferometric measurements. Observations are now dual-polarization, while other SMHB candidate sources, such as the centre of the Virgo cluster M87, have also been observed [230]. Current observations are being interpreted by a range of different models including the geometric crescent model proposed by Kamruddin & Dexter [228] (for example, figure 20), which qualitatively provides an excellent statistical description of the existing data. The first real resolved image of the shadow of the black hole at the centre of our Galaxy is expected in the next few years.

## 6. Concluding remarks

**Figure 21.**
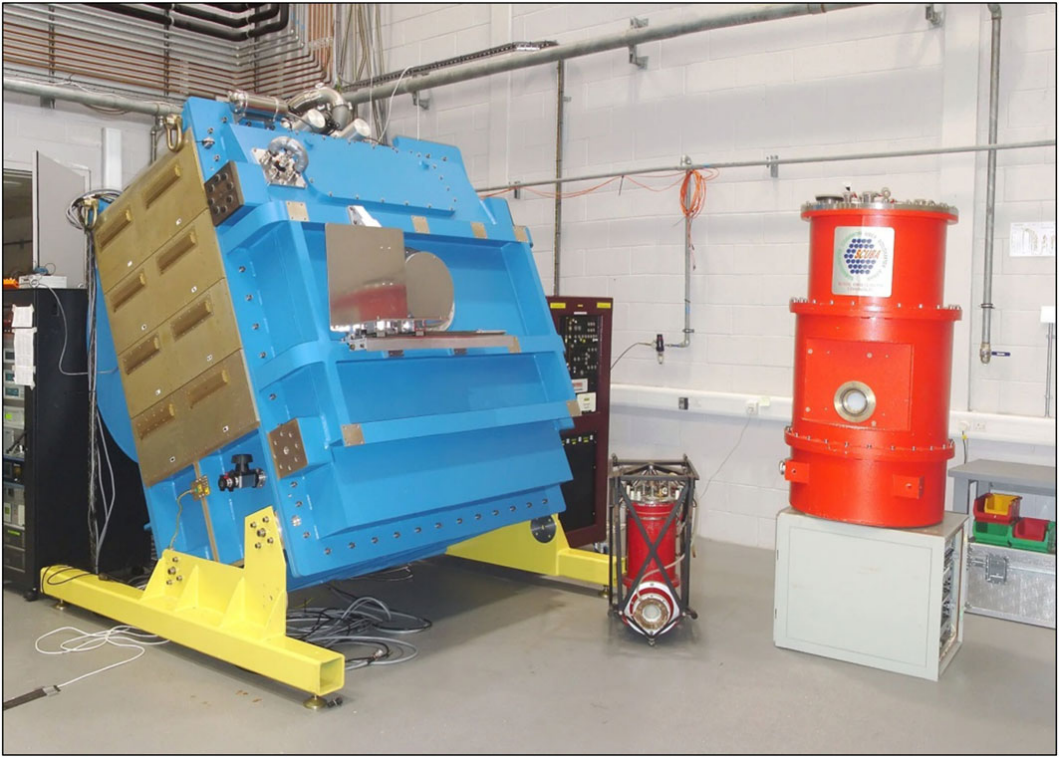
The JCMT continuum instruments together in the ROE Crawford laboratory, prior to the delivery of SCUBA-2 to JCMT (left) SCUBA-2 (10 000 pixels) (centre) UKT14 (1 pixel) and (right) SCUBA (128 pixels). SCUBA now resides in the National Museum of Scotland in Edinburgh. Photograph courtesy of the Royal Observatory Edinburgh.

**Figure 22.**
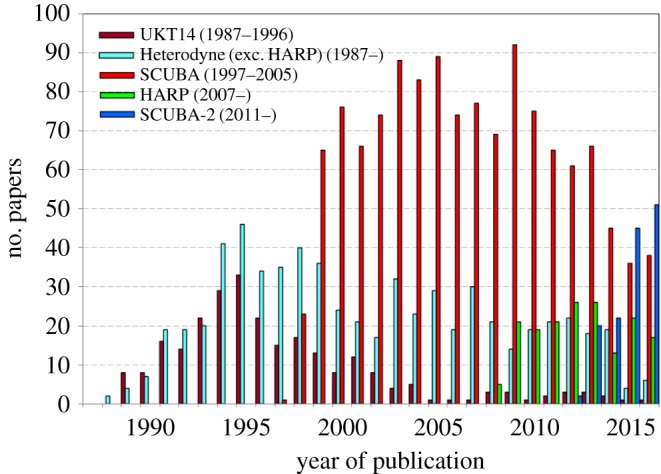
The number of peer-reviewed papers per year until the end of 2016 (information has been gathered from the SAO/NASA Astrophysics Data System). The dates in brackets refer to the operational period for the instrument (or range of instruments).

Looking back on the past history of the telescope, it is clear that the scientific impact relied on a number of timely technological innovations in new instrumentation (figure 21). Figure 22 shows the number of (peer-reviewed) papers over the past 30 years split between the three continuum instruments, the single/dual pixel heterodyne receivers and the HARP array. The plot is dominated by SCUBA with just over 50% of the total number of papers. In terms of an overall legacy, SCUBA had, without a doubt, one of the biggest impacts of any instrument built for an astronomical telescope. In the period 1997–2005, it revolutionized our knowledge in a number of areas of astronomy. In particular, it led to a major advance in the understanding of the astronomical origin questions: how planets, stars and galaxies form. SCUBA revealed discs of cold dust around nearby stars that are evidence that planet formation is ongoing or has already occurred. It has detected large numbers of young protostars and ‘pre-stellar cores’—objects on the brink of becoming stars, making possible the first statistical studies of the earliest stage of star formation. Finally, and perhaps most significantly, very shortly after the detection of a strong submillimetre background, SCUBA showed that this background is composed of high-redshift, ultraluminous, dusty galaxies. These galaxies have all the properties expected for elliptical galaxies in their formation stage, objects which have been looked for in vain for over a decade with optical telescopes. Indeed, the paper describing this seminal discovery now has over 1000 citations, making it the most cited scientific paper in the history of the JCMT.

Further evidence of the scientific impact of the JCMT has come from several analyses that ranked the productivity of telescopes in terms of three metrics: the numbers of papers published, the total number of citations and the number of citations per paper. A study by Trimble & Zaich [231] of 36 radio/millimetre/submillimetre telescopes for the year 2001 showed that the JCMT was the most successful with 21.1 citations per paper, almost twice as many as any other submillimetre telescope; the closest challenger being the IRAM interferometer with 19.7 citations per paper. This output continued at a similar level for a number of years [232] only diminishing slightly in terms of published paper count during the period 2010–2014 as SCUBA-2 was commissioned and the first results from this new instrument were forthcoming.

**Figure 23.**
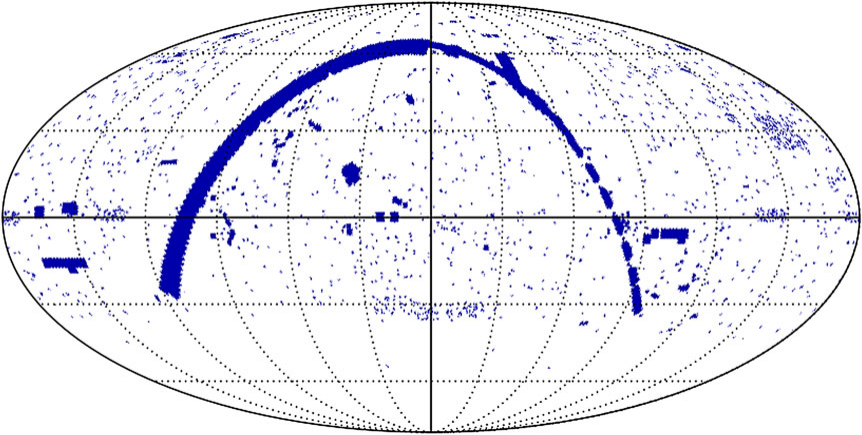
The areas of sky observed by SCUBA-2 up until December 2016, dominated by areas in or near the Galactic Plane. Figure courtesy of Graham Bell (EAO).

Now in its 30th year of operation, the JCMT continues to produce world-leading science. As of mid-2017, a number of new large scientific programmes have been awarded time on the telescope, including an extension to the BISTRO magnetic field survey of Gould Belt clouds, a new survey to resolve star formation in the Galactic Plane with HARP, a dust and gas survey of nearby evolved stars, and an extensive study of the Andromeda galaxy. The East Asian Observatory is now looking for opportunities to expand the capabilities of the telescope with a series of instrument upgrades over the next 5 years [233]. Of particular importance in this regard is to capitalize on the key strengths of single-aperture telescopes in an era that is becoming increasingly dominated by multi-element interferometers (such as the SMA and ALMA). There are initial design plans for a much larger (of order 100 pixel) 850 μm heterodyne array to replace the current HARP system. Despite the relatively large field-of-view, particularly compared to the predecessor instruments on the JCMT, SCUBA-2 has still only covered some 5.3% of the total sky visible from Mauna Kea (as shown in figure 23). New technologies are also emerging that could see SCUBA-2 upgraded with new detectors or indeed replaced by an even larger format (100 000+pixel) imaging camera [234]. Finally, a full replacement is planned for the Receiver A (operating at 1.3 mm) to allow science to continue even when the weather is not suitable for submillimetre observations. It is clear from these ambitious plans that the JCMT will have a bright and relevant future.

## Footnotes

1 This was due to the strongest winter El Niño event seen for many years in Hawaii. The next event of a similar magnitude did not occur until the winter of 2016/17.

## Data accessibility

This article has no additional data.
